# MicroRNA-Driven Developmental Remodeling in the Brain Distinguishes Humans from Other Primates

**DOI:** 10.1371/journal.pbio.1001214

**Published:** 2011-12-06

**Authors:** Mehmet Somel, Xiling Liu, Lin Tang, Zheng Yan, Haiyang Hu, Song Guo, Xi Jiang, Xiaoyu Zhang, Guohua Xu, Gangcai Xie, Na Li, Yuhui Hu, Wei Chen, Svante Pääbo, Philipp Khaitovich

**Affiliations:** 1 Key Laboratory of Computational Biology, CAS-MPG Partner Institute for Computational Biology, Chinese Academy of Sciences, Shanghai, China; 2 Max Planck Institute for Evolutionary Anthropology, Leipzig, Germany; 3 Graduate School of Chinese Academy of Sciences, Beijing, China; 4 Max Delbrück Center for Molecular Medicine, Berlin-Buch, Germany; 5 Max Planck Institute for Molecular Genetics, Berlin, Germany; Massey University, New Zealand

## Abstract

While multiple studies have reported the accelerated evolution of brain gene expression in the human lineage, the mechanisms underlying such changes are unknown. Here, we address this issue from a developmental perspective, by analyzing mRNA and microRNA (miRNA) expression in two brain regions within macaques, chimpanzees, and humans throughout their lifespan. We find that constitutive gene expression divergence (species differences independent of age) is comparable between humans and chimpanzees. However, humans display a 3–5 times faster evolutionary rate in divergence of developmental patterns, compared to chimpanzees. Such accelerated evolution of human brain developmental patterns (i) cannot be explained by life-history changes among species, (ii) is twice as pronounced in the prefrontal cortex than the cerebellum, (iii) preferentially affects neuron-related genes, and (iv) unlike constitutive divergence does not depend on *cis*-regulatory changes, but might be driven by human-specific changes in expression of *trans*-acting regulators. We show that developmental profiles of miRNAs, as well as their target genes, show the fastest rates of human-specific evolutionary change, and using a combination of computational and experimental methods, we identify miR-92a, miR-454, and miR-320b as possible regulators of human-specific neural development. Our results suggest that different mechanisms underlie adaptive and neutral transcriptome divergence, and that changes in the expression of a few key regulators may have been a major driving force behind rapid evolution of the human brain.

## Abstract 2

Comparison of human, chimpanzee, and macaque brain transcriptomes reveals a significant developmental remodeling in the human prefrontal cortex, potentially shaped by microRNA.

## Author Summary

Species evolution is often depicted as a slow and continuous process punctuated by rapid changes. One example of the latter is the evolution of human cognition–emergence of an exceedingly complex phenotype within a few million years. What genetic mechanisms might have driven this process? Nearly 40 years ago, it was proposed that human-specific gene expression changes, rather than changes in protein sequence, might underlie human cognitive evolution. Here we compare gene expression throughout postnatal brain development in humans, chimpanzees, and macaques. We find that simple changes in gene expression levels, plausibly driven by mutations in *cis*-regulatory elements, accumulate at similar rates in all three evolutionary lineages. What sharply distinguishes humans from other species is change in the timing and shape of developmental expression patterns. This is particularly pronounced in the prefrontal cortex, where 4-fold more genes show more human-specific developmental changes than chimpanzee-specific ones. Notably, our results indicate that this massive developmental remodeling of the human cortex, which affects hundreds of genes, might be driven by expression changes of only a few key regulators, such as microRNAs. Genes affected by this remodeling are preferentially associated with neural activity, thereby suggesting a link to the evolution of human cognition.

## Introduction

In multicellular organisms, the evolution of novel characteristics frequently involves gene expression change [1]. Nearly four decades ago, it was hypothesized that the evolution of the human brain could similarly be driven by expression changes [2]. In support of this, early comparative studies of adult human, chimpanzee, and macaque transcriptomes reported more human-specific expression changes than chimpanzee-specific changes in the prefrontal cortex (PFC) of the brain, but no such imbalance in other tissues such as blood, liver, or heart [3],[4]. Several studies further associated these expression differences with neuron-specific functions [5]–[7], and a recent analysis of the human, chimpanzee, and macaque PFC transcriptomes reported more human-specific than chimpanzee-specific changes in developmental timing [8].

Together, these studies suggest that the human brain transcriptome has evolved at an accelerated rate compared to that of the chimpanzee, possibly reflecting the accelerated rate of human cognitive evolution. Despite the attractiveness of this hypothesis, the ontogenetic and tissue-specific properties of this phenomenon have yet to be investigated. For instance, whether human brain transcriptome acceleration involves species differences that are constitutive across lifespan, or differences in how ontogenesis proceeds, is unclear. In addition, as most existing transcriptome comparisons studied a single brain region, we do not know whether different brain regions exhibit the same rate and kind of evolutionary acceleration. Finally, the molecular basis of human brain transcriptome acceleration, such as the contribution of *cis*- and *trans*-events [9], also remains unknown.

In the present study we analyzed prefrontal cortex (PFC) and cerebellar cortex (CBC) transcriptomes in humans, chimpanzees, and rhesus macaques of different ages. Both the PFC and CBC are brain regions potentially involved in human-specific behaviors. The cerebellum is important for motor function and memory and has been implicated in human language [10], while the PFC is associated with such functions as abstract thinking, planning, social intelligence, and working memory [11]. Using a number of datasets, we found that two types of divergence, both expression differences that are constitutive throughout lifespan as well as expression differences involving changes in developmental patterns, have contrasting functional and evolutionary properties. The accelerated evolution of human brain expression appears to mainly involve remodeling of developmental patterns, which may in turn be shaped by microRNA (miRNA) expression changes. Moreover, this acceleration is not uniform across the human brain and is particularly pronounced in the PFC.

## Results

**Figure 1:**
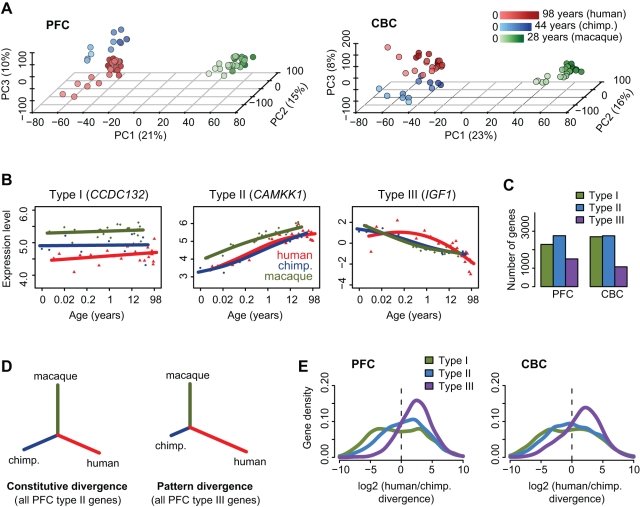
Gene expression variation in the PFC and CBC. (A) Principle components of the two brain regions' transcriptomes based on all expressed genes (CBC:12,853; PFC:12,447). Each individual is represented by a circle; colors represent species (red, human; blue, chimpanzee; green, macaque), and lighter shades indicate younger age. The proportion of variance explained by each component is shown in the axes labels. In both regions, the first component corresponds to macaque-hominid divergence, the second to age differences, while the third component (*z*-axis) separates humans and chimpanzees. (B) Examples of the three types of divergence in the PFC dataset. Each point represents an individual (red, human; blue, chimpanzee; green, macaque), while lines show cubic spline curves. The *x*-axis shows individuals' age in log_2_ scale. Gene names are shown on top of each panel. (C) Numbers of genes assigned to the three divergence types (Table S2). (D) Example neighbor-joining (NJ) representing constitutive and pattern divergence, based on expression profiles of PFC type II and type III genes. Trees were constructed per gene, using Euclidean distances between expression-age trajectories between each pair of species (Text S1). The figure reflects the mean divergence per gene set. (E) Distributions of log_2_-transformed human-chimpanzee branch ratios estimated from NJ trees across gene sets. Ratios <|10| are only shown for illustrative purposes. See Table S3 for additional analyses on the distributions.

Using Affymetrix microarrays we measured mRNA expression in the PFC and CBC of 12 to 26 individuals per species. The individual ages varied across a large portion of the species' lifespan, although most samples were chosen from the early postnatal period (Table S1; Figure S1A). Across the approximately 12,000 genes detected in either brain region, species differences could explain approximately 45% of total expression variation, while age could explain 30% (Figure S1B–C). Both effects were also manifest in principle component analyses (Figure 1A), and were highly reproducible upon comparison with published gene expression datasets (Figure S2).

In each brain region and species, we identified genes showing significant expression changes across the lifespan as well as genes showing significant expression divergence among species, using polynomial regression models and analysis of covariance, respectively (*p*<0.001, false discovery rate <10%). We then classified genes showing significant species divergence into the following three types (Figure 1B): type I, constant expression across lifespan; type II, variable expression across lifespan, no developmental pattern differences among species; type III, variable expression across lifespan and developmental pattern differences among species (developmental remodeling). We identified approximately 1,000–3,000 genes in each category, with type II being the most common and type III the rarest (Figure 1C, Table S2).

### Human Developmental Expression Patterns Show Accelerated Evolution

We estimated the expression divergence among species by calculating distances between species' expression-age trajectories and constructing neighbor joining trees (Materials and Methods). We found that the expression divergence in genes showing constant expression or no developmental pattern changes (type I and II), i.e. constitutive divergence, reflected the three species' known phylogenetic relationship (Figure 1D–E). Furthermore, human and chimpanzee branch lengths (which represent divergence on the respective lineages) for type I and type II genes were comparable, with average ratios of the human to chimpanzee branch lengths ranging between 1.0 and 1.6 (Table S3). Thus, the evolutionary rate of constitutive expression divergence roughly reflects the divergence between the two species from their common ancestor.

By contrast, when we studied species divergence in the *shape* of developmental trajectories among genes that underwent developmental remodeling (type III), we found markedly different results. In the PFC, the human branch was on average 5.2±0.7 times longer than the chimpanzee branch (Figure 1D–E, Table S3). The human branch was even 1.2±0.1 times longer than the “macaque” branch representing divergence between the common ancestor of humans and chimpanzees and contemporary macaques. If gene expression was evolving in a similar way to genomic sequence, the human branch would be expected to be approximately seven times shorter compared to this “macaque” branch [12]. In the CBC, the median human-chimpanzee branch length ratio was 3.5±0.5. Thus, in the CBC, developmental pattern divergence was also significantly greater on the human versus the chimpanzee evolutionary lineage, but to a significantly less extent than in the PFC (*p*<10^−8^). These results indicate that, while humans and chimpanzees evolved at similar rates with respect to constitutive expression differences, human developmental patterns have become highly differentiated from both chimpanzee and macaque developmental patterns, with 1.5- to 2-fold greater acceleration in the PFC.

We performed a number of analyses to test the validity of this conclusion, including (a) sub-sampling to equalize mean expression level distributions across the three gene types; (b) selecting the same number of individuals per species, with similar age-distributions across species' lifespan; (c) comparing the numbers of species-specific genes identified by the *F* test, as an alternative to our expression distance measure; (d) controlling for life-history differences among species, such as prolonged childhood and longer lifespan in humans [13]; and (e) repeating mRNA expression measurements in newborn and young adult pooled samples from each species by sequencing RNA with the Illumina platform (Text S1).

The excess of human developmental pattern divergence was found to be significant across all tests (Figure S3B–D, Table S3). Importantly, despite the limited sample size of the RNA-sequencing dataset, type III genes showed significantly more human-specific expression changes compared with the other two types in both brain regions (*p*<0.001; Figure S4) and this excess was significantly higher in the PFC than in the CBC (*p*<0.001). Considered together, these results indicate that the accelerated pace of developmental remodeling on the human evolutionary lineage is not caused by sampling or technical biases, and cannot be explained by life-history differences among species.

### Genes Showing Developmental Pattern Divergence Are Neuron-Related and Evolutionarily Conserved

**Figure 2:**
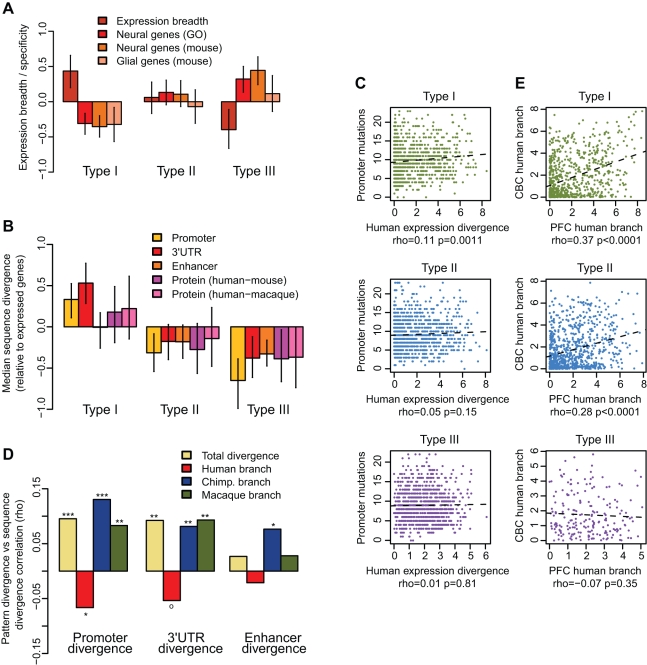
*Cis*-effects on expression divergence. (A) Mean expression breadth or nervous system specificity among divergent gene types in PFC, relative to all expressed genes in PFC. Expression breadth indicates the number of cell types in which a gene is expressed (Text S1). Neuron/glia-specificity measures were obtained from Gene Ontology [14] or a mouse experiment (mouse) [47]. Note that the mean specificity reflects the proportion of cell type-specific genes in a gene set. (B) Median sequence divergence among divergent gene types in the PFC, relative to all expressed genes in PFC. Promoter (±200 bp around the transcription start site), 3′UTR, and H3K4me1-enriched enhancer region divergence (Materials and Methods) divergence was estimated from pan-mammalian Phastcons scores [46]. Coding divergence reflects human-mouse or human-rhesus macaque dN/dS. Error bars represent 95% bootstrap intervals. Median values used as sequence divergence distributions are highly skewed. (C) Correlations between human-specific expression divergence in PFC (the human branch length based on an expression distance NJ tree) and human-specific promoter mutations (estimated from human, chimpanzee, and macaque alignments) across genes. For illustrative purposes, expression divergence measures were log-transformed. For full results see Table S5. (D) Correlation between species-specific type III expression divergence and pan-mammalian sequence divergence (negative Phastcons scores). “Total” denotes total expression divergence, or NJ tree size, per gene. ° *p*<0.10, * *p*<0.05, ** *p*<0.01; based on Spearman correlation tests. The same calculation based on alternative region definitions is shown in Figure S9. (E) Correlations between species-specific expression divergence in the PFC and CBC among the three gene sets in humans, using genes showing the same divergence type in both regions. See Figure S6 for chimpanzee and macaque data. To control for the influence of expression level on conservation or expression breadth, we used gene subsets with equalized mean expression level distributions (panels A–D).

Could the accelerated evolution of brain developmental patterns on the human lineage reflect adaptive changes underlying human cognitive evolution? To address this possibility, we investigated the functional properties of type I, II, and III genes. Using the Gene Ontology [14] and Kyoto Encyclopedia of Genes and Genomes (KEGG) databases [15], we detected significant enrichment of PFC type III genes in neuron-related functional processes, including “neuroactive ligand-receptor interaction” and “behavior” (Table S4, *p*<0.05). CBC type III genes did not show significant associations with neural functions, but were enriched in more general processes such as “response to hormone stimulus” and “response to hypoxia,” while type I genes were enriched in mRNA processing and splicing-related processes in both brain regions. Type II genes showed no significant functional associations. Further, with respect to expression breadth, type III genes showed the greatest tissue-specificity, and significantly overlapped with neuron-related genes, but not glia-enriched genes (Figure 2A, Figure S5). In contrast, expression breadth was significantly above the genome average for type I genes and close to average for type II genes, with no significant enrichment among neuron- or glia-specific genes for either gene type (Figure 2A). Notably, PFC genes associated with neural function through Gene Ontology had ∼1.5 times larger human-chimpanzee branch ratios than non-neural type III genes (*p*<0.002). In accordance with the general conservation of neuron-related genes [16], regulatory and coding sequences of type III genes, but not the other gene sets, were significantly conserved compared to all genes expressed in the brain (Figure 2B). Overall, developmental remodeling in the PFC showed a clear association with brain-specific functional processes, while no such association could be detected for the other divergence types.

### *Cis*-Regulation of Gene Expression Divergence

We next examined the possible mechanisms underlying PFC expression divergence for the three gene types. Expression divergence could be caused either by *cis*-changes that accumulate in regulatory regions over time and tend to affect each gene independently, or by altered expression, or function, of a few *trans*-regulators affecting the expression of hundreds of genes in a coordinated manner. To determine which mechanism played the more dominant role in human expression evolution, we first tested the correlation between gene expression divergence and sequence divergence in *cis*-regulatory regions on the human, chimpanzee, and macaque evolutionary lineages. To this end we analyzed the proximal promoter, the 3′UTR, and putative enhancer regions, using Ensembl [17] gene annotation, as well as regions defined using histone modification data derived from human cell lines [18] or human and mouse brain tissue (Materials and Methods) [19],[20].

On the macaque lineage, which contained the greatest number of sequence changes, we detected a significant positive correlation between promoter sequence divergence and expression divergence for the type I and type II genes (rho∼0.10, *p*<0.05, Table S5). Similarly, we detected a significant and positive correlation between human-specific promoter sequence divergence and human-specific expression divergence across the type I genes, and a positive correlation trend for type II genes (Figure 2C). However, we found no significant correlations between promoter divergence and expression divergence on the chimpanzee lineage, or for the type III genes on any lineage (Table S5). This observation may be due to the paucity of species-specific mutations resulting in insufficient power in our tests. To overcome this limitation, we tested correlations between regulatory region sequence divergence across mammalian species (pan-mammalian) and species-specific expression divergence. For type III genes, both macaque- and chimpanzee-specific expression divergence correlated significantly and positively with pan-mammalian regulatory sequence divergence. Notably, human-specific expression divergence did not correlate positively with pan-mammalian regulatory sequence divergence, but showed a significant negative correlation, albeit marginally (Figure 2D). In other words, whereas regulatory regions of type III genes diverging on the macaque and chimpanzee lineages are less conserved, those diverging on the human lineage are more conserved. This trend of positive association between type III expression divergence and sequence divergence in chimpanzees and macaques, and negative association in humans, was also observed in 3′UTR and enhancer regions (Figure 2D, Table S5). Thus, the expression divergence of type I and II genes on all evolutionary lineages, as well as the divergence of type III genes on the chimpanzee and macaque lineages, may well be linked to sequence divergence in *cis*-regulatory regions. By contrast, type III expression divergence (developmental remodeling) on the human evolutionary lineage does not reveal any such trend. This suggests that mutation accumulation in *cis*-regulatory regions might not have been the major contributor to the extensive remodeling of the developmental expression patterns observed in the human PFC.

The *cis*- and *trans*-expression divergence mechanisms can also be indirectly discerned through their tissue-specificity. While mutations in *cis*-regions tend to affect multiple tissues, *trans*-factor-driven changes tend to be more tissue-specific [21]. Indeed, we found constitutive divergence (type I and II genes) to be highly correlated between PFC and CBC in all three species, while developmental pattern divergence (type III genes) showed no such correlation (Figure 2E, Figure S6). This result further supports the notion that constitutive divergence (type I and II) is driven largely by changes in *cis*-elements, but this is not the case for developmental pattern divergence in the human PFC.

### MicroRNA Contribute to Human-Specific Developmental Pattern Divergence

**Figure 3:**
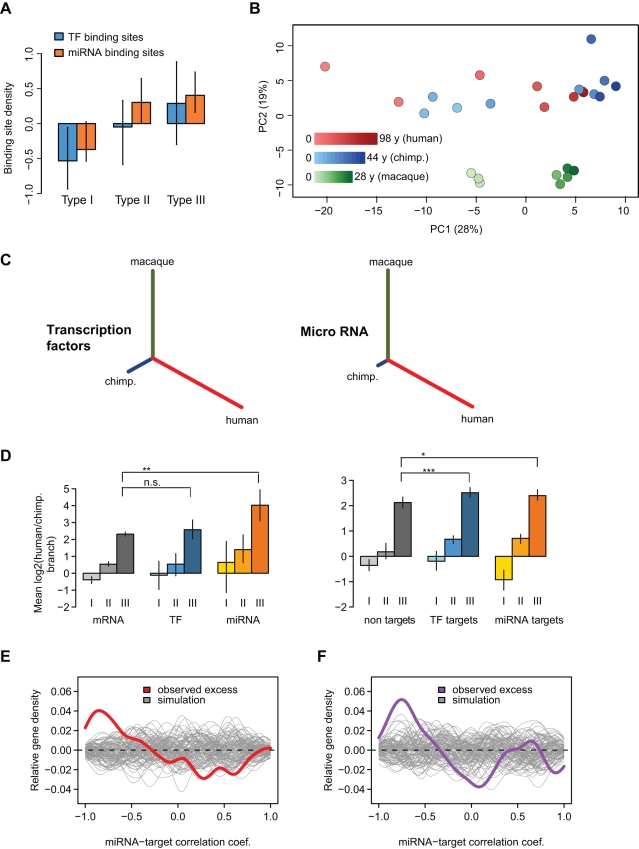
*Trans*-effects on developmental pattern divergence in the PFC. (A) Median density of conserved TF and miRNA binding sites (BS) among divergent gene types in the PFC, relative to all genes with relevant information. TFBS were identified in the proximal promoter and miRNA-BS in the 3′UTR divergence using Transfac [22] and TargetScan databases [23], respectively. Error bars represent 95% bootstrap intervals. To control for influence of expression levels, for each type we used gene subsets with equalized mean expression level distributions. We further removed the overall sequence conservation effect on BS density using linear regressions (Text S1). (B) Principle components of the PFC miRNA transcriptome based on 198 detected miRNAs. Proportion of variance explained by each component is shown in the axes labels in parentheses. Each individual is represented by a circle; colors represent species (red, human; blue, chimpanzee; green, macaque), while lighter shades correspond to younger age. (C) Mean NJ trees for 68 TFs and 19 miRNA of type III, as in Figure 1D. (D) Median log_2_ human-chimpanzee branch ratios among genes showing the three types of divergence (I, II, and III), for mRNA, TFs, and miRNA, and for genes either targeted by miRNA (miRNA-tar.) or TFs (TF-tar.) of the same type of divergence, and all other genes (non-tar.). Error bars show 95% bootstrap confidence intervals. *** *p*<0.001, ** *p*<0.01, * *p*<0.05, n.s., not significant; based on Wilcoxon tests. (E) Excess of negative correlations between miRNAs and their predicted targets. The colored line shows the distribution of correlations between miRNA-target pairs relative to miRNAs and non-target pairs (observed excess). The grey lines depict the random expectation estimated by 100 permutations of miRNA-non-target pairs. See Table S6 for similar analyses including TFs and CBC. (F) Same as panel E, but here we use species differences (across expression-age trajectories) to calculate regulator-target correlations.

We hypothesized that type III divergence and, in particular, human-specific developmental pattern divergence in the PFC could be driven by *trans*-factors. In support of this notion, we found that type III genes, in comparison to the other gene sets, carry a higher density of predicted miRNA and transcription factor (TF) binding sites [22],[23] in their regulatory regions, even when accounting for conservation level differences among genes (Figure 3A). We next considered whether *trans*-regulators, specifically TFs and miRNAs, also show an increased divergence in developmental patterns on the human lineage. For this, we used expression profiles for 105 TFs from mRNA microarray data that had target prediction information. In addition, we measured expression of ∼200 miRNAs in the PFC and CBC of humans, chimpanzees, and rhesus macaques, in 7 to 12 individuals of different age per species, using Agilent microarrays (Figure 3B, Figure S7A). We found that although TFs showed comparable human divergence to other genes, for 19 PFC-expressed type III miRNAs the median branch length was 24 times longer for humans than for chimpanzees (Figure 3C–D). This effect was stable after correcting for life-history differences among species (Table S3). By contrast, the median human-chimpanzee branch ratio based on 15 CBC-expressed type III miRNAs was found to equal 3.9. Thus, miRNAs in the PFC show an even greater excess of human-specific developmental remodeling than other transcripts. Despite the limited power of available target prediction algorithms, we also detected greater human-chimpanzee branch ratios among predicted targets of type III TF and type III miRNAs compared to non-target genes (*p*<0.05; Figure 3D). Hence, evolutionary changes in these *trans*-regulator expression trajectories could underlie the excess of developmental pattern divergence in the human PFC.

Focusing on PFC type III genes, we next directly tested possible associations between regulators and predicted target genes. We found a significant excess of negative correlations between miRNAs and their predicted targets' expression profiles (*r*<−0.9, *p*<10^−6^; Figure 3E), consistent with the inhibitory role of miRNAs on expression. For TFs, we observed a modest excess of positive correlations (*r*>0.9, *p* = 0.08; Table S6). As variation in expression profiles reflects both age effects and species differences, we also calculated regulator-target correlations based only on species' expression differences across the lifespan. Again, in the PFC, species differences in miRNA expression showed a significantly negative association with differences in their predicted targets' profiles (Figure 3F, Table S6).

To estimate the effect of miRNA regulation on developmental pattern divergence in the PFC, we searched for miRNAs with significant target site enrichment among PFC type III genes. Screening all 157 expressed miRNAs using TargetScan predictions [23], we detected 39 miRNAs with significantly more predicted targets among PFC type III genes than among other gene sets (*p*<0.05). Importantly, detection of only five miRNAs would be expected by chance (permutation test *p*<0.001). Of the 39 miRNAs identified, 12 showed an excess of negative correlations with their type III targets, compared to the 118 miRNAs with no target enrichment in PFC type III genes (one-sided binomial test *p*<0.05; Table S7, Table S8). The 140 target genes associated with these miRNAs constitute approximately 10% of the developmental remodeling events identified in the PFC. Notably, the majority of these miRNAs (90%), as well as their targets (89%), showed more divergence in humans than chimpanzees.

### Experimental Validation of miRNA-Target Relationships

**Figure 4:**
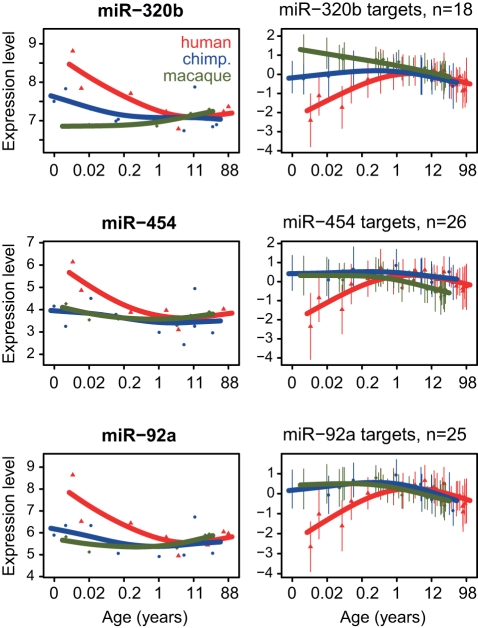
Examples of candidate regulators of PFC developmental remodeling (left panels) and their predicted and negatively correlated targets (right panels). The three miRNAs were chosen based on predicted target enrichment among type III genes and an excess of negative correlations with predicted targets, using both expression profiles and species differences (Text S1, Table S8). Each point represents an individual (red, human; blue, chimpanzee; green, macaque), and lines show cubic spline curves. The *x*-axis shows individuals' age in log_2_ scale. In the target profile plots, the *y*-axis shows the mean standardized expression level among target genes, while error bars indicate one standard deviation from the mean.

To validate some of the predicted miRNA regulatory effects, we conducted an additional set of experiments with the miRNAs miR-92a, miR-454, and miR-320b. These miRNAs showed not only significant enrichment of predicted targets among type III genes, but also the strongest excess of negative correlations with their predicted targets in our analyses (Figure 4). At the same time, even though these miRNAs have been associated with malignancies [24] and angiogenesis [25] in the brain, to our knowledge, none of them have yet been implicated in brain developmental regulation.

**Figure 5:**
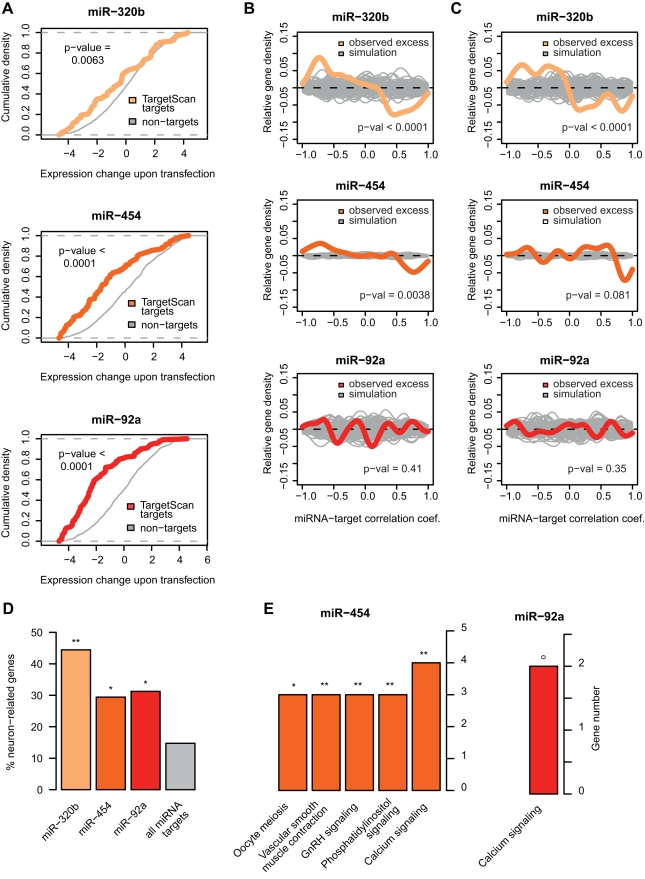
Regulatory effects of miRNA tested in cell lines. (A) Gene expression shifts in miRNA-transfected cell lines. The colored and grey lines show the cumulative density (*y*-axis) of the expression change magnitude upon transfection (*x*-axis), for target genes predicted by TargetScan among PFC type III genes, and all other type III genes, respectively. The *x*-axis is calculated as the expression level difference between miRNA-transfected and negative control-transfected neuroblastoma cell lines, per gene (Materials and Methods). The *p* values were calculated by one-sided Wilcoxon tests. (B) Excess of negative correlations between miRNAs and their targets identified based on inhibition in the cell line experiments. The colored line shows the distribution of correlations between miRNA-target pairs relative to miRNAs-non-target pairs (observed excess). The grey lines depict the random expectation estimated by 100 permutations of miRNA-non-target pairs. This analysis does not include information from TargetScan predictions. The *p* values were calculated by one-sided Wilcoxon tests. (C) Same as panel C, but using species differences (across expression-age trajectories) to calculate regulator-target correlations. Panels B–C are analogous to Figure 3E–F. (D) Proportion of neuron-related genes, based on Gene Ontology [14], among the three miRNAs' verified gene sets. The fourth column indicates the proportion of neuron-related genes among all PFC-expressed miRNA target genes, which was used as background in the enrichment tests. ** *p*<0.01, * *p*<0.05, ° *p*<0.10; based on one-sided Wilcoxon tests. (E) KEGG pathways enriched among verified targets of miR-454 and miR-92a. ** *p*<0.01, * *p*<0.05, ° *p*<0.10; based on one-sided hypergeometric tests, Bonferroni-corrected.

First, we directly tested predicted miRNA-target associations by transfecting two human neuroblastoma cell lines with miRNA constructs containing mature sequences for the three miRNAs (Materials and Methods). The regulatory effects were determined by comparing gene expression in cell lines transfected with miRNA constructs or transfected with negative controls, using Affymetrix microarrays. In all cases, we detected a strong inhibitory effect of the miRNA on target genes predicted by TargetScan [23], compared to non-target genes (Figure 5A, Figure S8). We also found that genes inhibited in the miRNA transfection experiment, irrespective of TargetScan annotation, showed a trend of negative associations with miRNA expression in our time series dataset (Figure 5B–C). Thus, based on miRNA targets experimentally identified in neuroblastoma cell lines, we confirmed the validity of TargetScan predictions used in our computational analysis, and directly demonstrated negative associations between miRNA expression and the expression of their type III target genes.

We then asked whether the verified type III target genes of the three miRNAs, i.e. the targets predicted by TargetScan that showed negative correlations in our time series and expression inhibition in the transfection experiments, might also be associated with particular functions and/or cell types. We found that targets of all three miRNAs were neuron-related when compared to other miRNA targets (*p*<0.05 in each case, Figure 5D). Furthermore, targets of two out of the three miRNAs tested, miR-454 (*n* = 18) and miR-92a (*n* = 20), showed enrichment in the same KEGG term: “calcium signaling” pathway (Figure 5E, Table S9). This is noteworthy, since miR-454 and miR-92a do not share common targets except for one gene, and finding such a functional overlap between two such miRNA target sets is unlikely (*p* = 0.021). Intriguingly, calcium signaling is a major biological processes involved in neuronal plasticity [26],[27], implying a role of miR-92a and miR-454 in regulating genes associated with neurodevelopmental plasticity.

**Figure 6:**
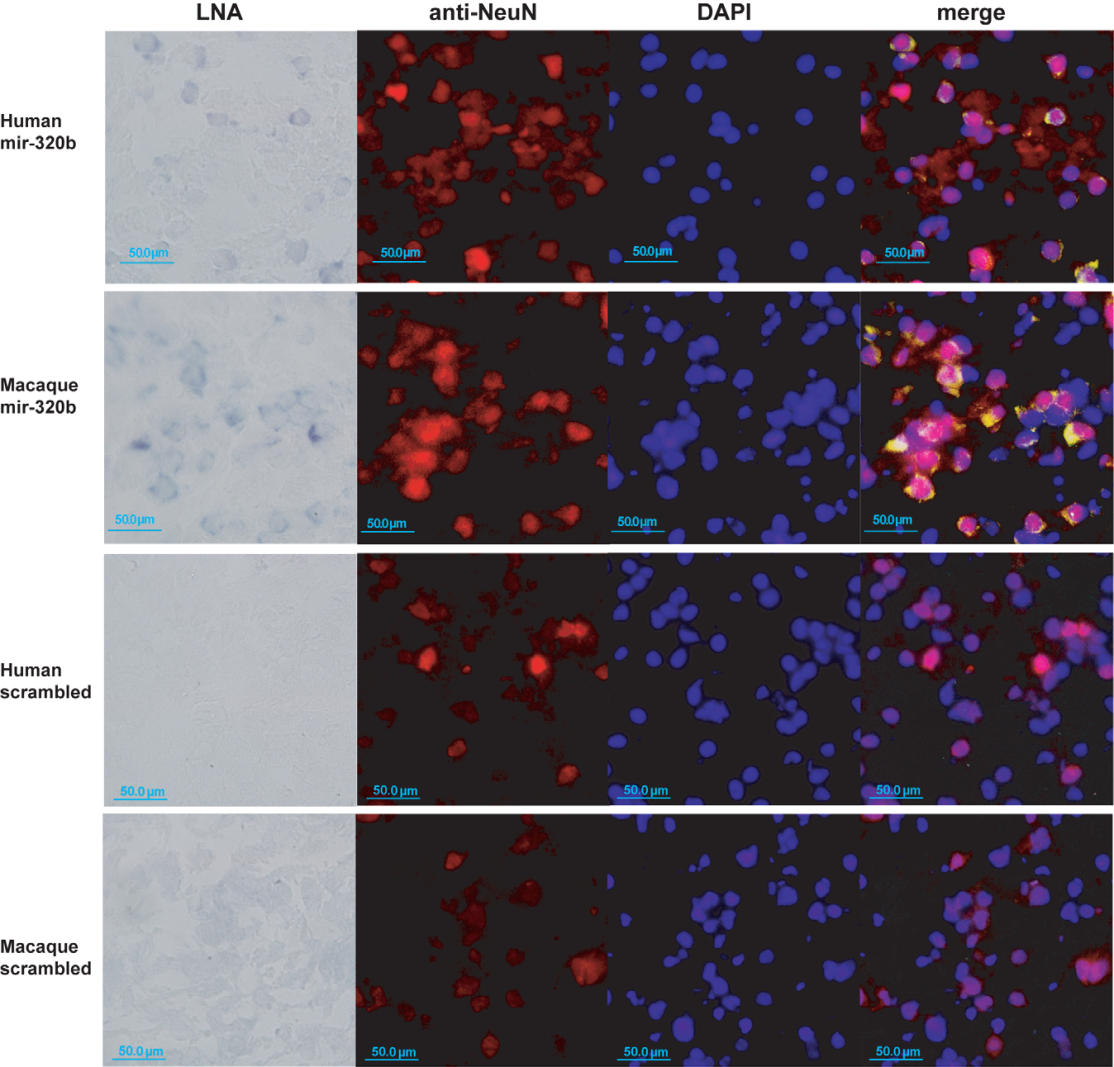
In situ staining of miR-320b in prefrontal cortex. First row: Human newborn prefrontal cortex sections were hybridized with miR-320b LNA-probes (far left); anti-NeuN antibodies staining neuron nuclei (center left) and DAPI staining DNA (center right); and a merged image showing LNA probes in yellow (far right). The LNA picture was taken under bright field at 40× magnification, and the DAPI and anti-NeuN pictures used the fluorescent channel. For the merged image the LNA signal was modified to a green color scale. Second row: Rhesus macaque prefrontal cortex sections processed and displayed in the same way as human. Third and fourth rows: Human and rhesus macaque sections were treated the same way as described, but using LNA probes with scrambled miRNA sequences as a negative control. The scale bar indicates 50.0 µm. See Materials and Methods for details.

In a second experiment, we investigated the histological expression patterns of miR-320b, the miRNA showing the strongest enrichment in neuron-related genes (Figure 5D). By means of in situ hybridizations with an LNA-containing probe complementary to the mature sequence of this miRNA in human and macaque prefrontal cortex sections (Materials and Methods), we found that miR-320b preferentially co-localized with neurons in both species (Figure 6). These results are consistent with the proposed regulatory relationship between this miRNA and its neuron-related target genes.

## Discussion

### Constitutive and Developmental Pattern Divergence Exhibit Distinct Characteristics

Our analysis marks constitutive divergence (represented by type I and II genes) as the most dominant form of expression difference among species. In the primate brain, 85%–90% of genes showing significant expression divergence between humans and chimpanzees fall within this category. Expanding our analysis to a published brain transcriptome time series from two mouse species (*Mus musculus* and *Mus spretus*) [8], 99% of genes with significant expression divergence can be classified as showing constitutive expression divergence.

Constitutive expression divergence affects the two brain regions analyzed in a similar manner. Genes showing constitutive divergence tend to have high expression breadth, show no association with tissue-specific functions, and their regulatory sequences evolve as fast as, or faster than, those of other genes. Notably, humans and chimpanzees show approximately equal levels of constitutive divergence in both brain regions studied. Thus, while some constitutive expression differences may underlie adaptive changes in primate brain evolution, the majority could represent transcriptomic drift [28], driven by mutations in *cis*-regulatory regions, and affecting multiple tissues. This result supports a previously proposed transcriptome evolution model, in which a large portion of expression divergence among species is shaped by *cis*-changes [29].

Developmental pattern divergence (type III genes), as identified in this study, shows a very different evolutionary mode. First, this is the least frequent divergence type, with less than 15% of genes differentially expressed between human, chimpanzee, and macaque brains showing developmental pattern differences. This might be expected given the general conservation of developmental expression trajectories among primates (Figure S2) [8]. Second, developmental pattern divergence differs substantially between the two brain regions analyzed, PFC and CBC. Third, developmental pattern differences found in the PFC are associated with neurons and neuronal functions. Fourth, in contrast to type I and II genes, type III genes have highly conserved regulatory and coding sequences. Furthermore, human-specific developmental pattern divergence in PFC shows negative correlation with *cis*-regulatory changes. This is striking, given that evolutionary expression divergence, including both quantitative and developmental pattern differences, is generally positively associated with regulatory and/or coding divergence [9],[29]–[32]. These results support a scenario, where tissue-specific changes in the expression of *trans*-regulators, such as miRNA, rather than sequence changes in *cis*-regulatory regions, are the driving force underlying developmental remodeling across hundreds of genes.

### A Role for miRNAs in Shaping Developmental Divergence among Species

While divergence in transcription factor expression and activity has been shown to underlie evolutionary divergence in various contexts (e.g., [33],[34]), relatively little is known about the role of miRNA in transcriptome evolution. It is, therefore, of note that the strongest *trans*-regulatory signal associated with developmental pattern divergence in the primate brain could be associated with miRNAs rather than with TFs. The regulatory effects of the top three miRNAs identified by computational predictions could be further supported by cell line transfections and in situ hybridization experiments. Our analysis further implicates two of these miRNAs in the regulation of genes involved in the calcium signaling pathway in neurons. Given the role played by calcium signaling in neuronal plasticity [26],[27], this result raises the possibility that the observed human-specific developmental expression patterns in the PFC could underlie human-specific cognitive functions such as learning and memory. It would be interesting to test miR-454 and miR-92a as potential regulators of neurodevelopmental plasticity in vivo.

Another noteworthy aspect of the miRNA experimental results is the spatial expression patterns identified by in situ hybridization. These indicate substantial variation among neurons in the miRNAs' expression (Figure 6), raising the possibility that the identified miRNAs are specific to different neuron subpopulations. Differential miRNA activity among species may thus contribute to brain organization differences, which have been suggested as a major distinction between human and chimpanzee brains [35].

Meanwhile, our work has a number of limitations. First, as our main analysis is based on human microarrays, we exclude any probes that do not match each of the three species' genomes perfectly and uniquely (Materials and Methods). Thus, we ignore regions that show copy number variation among species, or are species-specific. Likewise, our analysis is restricted to regulators conserved across the three species. This is important, because fast evolving or human-specific genes, TFs [36], or miRNAs [37] may have significant contributions to the evolution of primate brain development and species divergence [38]. Second, when analyzing the effects of *cis*-regulation on expression divergence, we are constrained by the limited annotation of *cis*-regulatory regions, and it is conceivable that mutations in a yet unidentified class of neural enhancers drive a substantial portion of developmental pattern divergence in humans. Third, our study is restricted to species differences in postnatal development. This is a critical period in brain ontogeny, when multiple processes, including synaptic maturation and myelination, take place [39]. Accordingly, expression differences in this period can readily generate phenotypic differences between species. Meanwhile, human-specific expression changes in *earlier* developmental stages could have at least as strong effects on the brain phenotype [40]. The evolution of fetal brain gene expression remains to be studied.

Despite these limitations, our results extend earlier observations on accelerated evolution of gene expression in the human brain. Specifically, we show that compared with chimpanzees and macaques, humans exhibit extreme developmental remodeling in the PFC and, to a lesser extent, in the CBC. Accelerated divergence of human developmental patterns cannot be explained by neutral drift, and cannot be attributed to life-history differences among species. Mechanistically, these human-specific changes appear driven by changes in expression of *trans*-acting regulators, and specifically miRNA, rather than accumulation of *cis*-regulatory mutations. Assuming a link between gene expression pattern divergence and phenotypic changes in the human brain, these results suggest that our cognitive abilities might be traced to a handful of key regulatory changes that remodeled cortical development.

## Materials and Methods

### Sample Collection

We collected prefrontal cortex (PFC) and cerebellar cortex (CBC) samples from postmortem brains of humans, chimpanzees, and rhesus macaque individuals of different ages (Table S1). Human samples were obtained from the NICHD Brain and Tissue Bank for Developmental Disorders at the University of Maryland (USA), the Netherlands Brain Bank (Amsterdam, the Netherlands), and the Chinese Brain Bank Center (Wuhan, China); chimpanzee samples from the Yerkes Primate Center (Georgia, USA), the Anthropological Institute & Museum of the University of Zürich-Irchel (Switzerland), and the Biomedical Primate Research Centre (the Netherlands); and the macaque samples from the Suzhou Experimental Animal Center (China). All subjects were healthy and had suffered sudden deaths. Informed consent for use of human tissues for research was obtained in writing from all donors or their next of kin. All non-human primates used in this study suffered sudden deaths for reasons other than their participation in this study, and without any relation to the tissue used. Detailed information on sample sources can be found in Text S1.

### Gene Expression Experiments

For details of experimental methods and quality assessment, see Text S1. For mRNA profiling we used postmortem brains of a total of 33 human (aged 0–98 years), 14 chimpanzee (aged 0–44 years), and 34 rhesus macaque individuals (aged 0–28 years). Total RNA extracted from 100 mg of dissected CBC and PFC samples (humans, *n* = 22/23; chimpanzees, *n* = 12/12; macaques, *n* = 24/26, respectively; Table S1) was hybridized to Affymetrix Gene1.0 ST human microarrays following standard protocols. Data analysis was conducted using probes matching all three species' genomes perfectly and uniquely, as determined using BLAT [41]. MicroRNA expression was measured among 14 humans, 11 chimpanzees, and 8 macaques using the Agilent Human microRNA Microarray (v3), containing probes for 866 human microRNAs. As the miRNA microarray probes are designed based on human mature miRNA sequences, we removed miRNA showing any sequence difference in their mature sequences, using BLAT [41] and aligning mapped sequences by ClustalW2 (http://www.ebi.ac.uk/Tools/msa/clustalw2). Five hundred human miRNA showing 100% conservation across the three species were included. Only samples with >200 detected miRNA were used. In total, 8 human, 11 chimpanzee, and 8 macaque PFC samples and 12 human, 7 chimpanzee, and 7 macaque CBC samples were retained. Only miRNAs that could be detected in >50% of samples per species were used. All microarray gene expression datasets were log_2_ transformed and quantile normalized. RNA-sequencing was conducted in pooled samples from newborn and young adults from the three species in each brain region, using the Illumina Genome Analyzer II system. Chimpanzee and rhesus macaque gene annotations were constructed from human gene annotation using the UCSC “Liftover” tool (http://www.genome.ucsc.edu/cgi-bin/hgLiftOver). Gene annotations were filtered to only include transcripts with a similar size in all three species. Transcript expression levels were calculated using “Cufflinks” (http://cufflinks.cbcb.umd.edu/), based on “Tophat” mapping of conserved exons [42]. The mRNA microarray datasets are available at NCBI GEO (http://www.ncbi.nlm.nih.gov/geo/) (accession number GSE22570). The miRNA datasets have been submitted to NCBI GEO (accession number GSE29356) and are available in processed form at the following locations for PFC and CBC, respectively: http://www.picb.ac.cn/Comparative/data_methods/data_ms_mirna_age_pfc_2011.txt and http://www.picb.ac.cn/Comparative/data_methods/data_ms_mirna_age_cbc_2011.txt.

The processed RNA-sequencing datasets are available at the following locations for PFC and CBC, respectively: http://www.picb.ac.cn/Comparative/data_methods/age_divergence_2010/rnaseq_hcm_PFC1.txt and http://www.picb.ac.cn/Comparative/data_methods/age_divergence_2010/rnaseq_hcm_CBC1.txt.

### Statistical Tests

For detailed descriptions of the tests applied, justifications of statistics, and detailed results, see Text S1. We used polynomial regression to model expression changes with age (age-test) and analysis of covariance (ANCOVA) to model species differences (differential expression test). In both tests, we fit data to families of models including linear, quadratic, and/or cubic terms [8],[43]. The *F* test was used to assess significance. We used a log_2_-age scale to model expression changes with age. When indicated, we also used ages transformed using linear models of life-history landmarks in order to normalize life-history differences among species. For identifying developmental pattern divergence among species, we used the ANCOVA test on datasets where mean (constitutive) expression differences among species had been subtracted, such that the only remaining difference was differences in trajectory shapes. False discovery rates were estimated by permuting individuals' age or species identity. Confidence ranges for the median ratio reported in the text indicate 95% bootstrap confidence intervals. In the RNA-sequencing experiment, genes were binary coded to show higher divergence in human or chimpanzee lineages; we therefore used the binomial test to compare distributions between gene sets. We used the Wilcoxon signed rank test for comparing log_2_-transformed human-to-chimpanzee branch length ratio distributions between the two gene sets, testing for a median >0 in the distribution. For testing enrichment of a gene set in functional categories or among targets of regulators, we used the hypergeometric test. In functional analyses the *p* values were corrected using the Bonferroni method. We used Spearman rank correlation for comparing sequence and expression divergence estimates. For identifying candidate miRNAs regulating specific gene expression patterns, we used the approach described in [44]. Briefly, we first test an association between targets of each miRNA and a gene set showing a particular gene expression pattern, using the hypergeometric test. Second, we test if the significantly enriched miRNAs show a higher frequency of negative correlations (Pearson *r*<−0.75) with their targets in the gene set, compared to the mean frequency for miRNAs without enrichment in the gene set. The frequencies were compared using the binomial test. The negative correlations were calculated by (a) using interpolated points from spline curves fit to the miRNA and mRNA expression profiles, using all three species together, and (b) calculating differences between species' curves at interpolated points, for miRNA and mRNA, using all three pairs of differences (Text S1). For comparing expression change distributions and correlation distributions for Figure 5A–C, we used one-sided Wilcoxon tests.

### Definition of the Three Divergence Types

Type I, constitutive divergence among constant genes: genes showing constant expression (age-test *p*>0.01 in all three species) and constitutive divergence across lifespan (differential expression test *p*<0.001 in at least one pair of species). Type II, constitutive divergence among developmental genes: genes showing age-related change (age-test *p*<0.001 in minimum one species) and constitutive divergence across lifespan (differential expression test *p*<0.001 in at least one pair of species), but no evidence for developmental pattern divergence (pattern divergence test *p*>0.01 in all species comparisons). Type III, divergence in developmental patterns: genes showing developmental regulation (age-test *p*<0.001 in at least one species) and differences in developmental expression patterns among species (both differential expression test and pattern divergence test *p*<0.001 in at least one pair of species).

### Expression Divergence Estimates

To estimate expression divergence, we first reduced the influence of individual variation within a species by fitting expression trajectories to each species' data points, for each gene independently. We next calculated Euclidean distances among these trajectories and constructed neighbor joining trees based on these distances (Text S1). Finally, expression divergence on each lineage was estimated as the length of the corresponding neighbor joining tree branch. Importantly, for type III genes we removed average expression level differences among species from the expression divergence calculation. Therefore, our estimates of expression divergence for type III genes purely reflect developmental pattern differences among species (Figure 1B).

### Processing of TF and miRNA Datasets

The TF expression profiles were obtained from the Affymetrix arrays. For the average TF expression divergence patterns shown in Figure 3C–D, we used all 1,071 genes annotated within the Gene Ontology category “transcription factor activity” (GO:0003700). Using alternative definitions, such as the list described in [45], or restricting the analysis to TFs annotated in TRANSFAC yielded similar results (unpublished data). miRNA profiles were measured using Agilent arrays and processed as described above.

### TF and miRNA Target Predictions

The procedure to define conserved human miRNA and TFBS target sites is described in [44]. For miRNA target prediction we used the Conserved Site Context Score Table from TargetScan (v5.0) [23]. We identified conserved TF binding sites (TFBS) in proximal promoters (±2,000 b.p. around the transcription start site) for each Ensembl human gene (v54), using the Match algorithm which uses binding site matrices from the TRANSFAC database (v11.2) [22]. We chose TFBS with average Phastcons scores ≥0.6 using the 17-way vertebrate Conserved Element Table [46]. TFBS were linked to transcription factor genes using two paths described in Text S1, which resulted in 426 TFBS mapped to ≥1 gene that were annotated with “transcription factor activity” (GO:0003700). For the analysis presented in Figure 3A, we removed the overall sequence conservation effect on BS density, using residuals from linear regressions between TFBS/miRNA-BS density and promoter/3′UTR mean Phastcons scores, respectively.

### Functional Analyses

For functional pathway analyses, we used Kyoto Encyclopedia of Genes and Genomes (KEGG) pathway annotation [15] and Gene Ontology (GO) annotation [14] for humans. To estimate expression breadth per gene we used cell type information from the GNF dataset available from Ensembl (v54) [17]. For estimating the neural-enrichment per gene, we used a mouse cell type-specific gene expression experiment with expression profiles containing the terms “neuron,” “axon,” and “synapse” [47]. In addition, we used all genes containing the terms “neuron,” “axon,” and “synapse” in GO. Data were preprocessed as described in [44]. See Text S1 for details.

### Sequence Divergence Estimates

For estimating pan-mammalian regulatory site conservation, we used the mean Phastcons 18-way Placental Mammal Conservation Track [46] values. Mean scores were calculated for (a) proximal promoters and (b) 3′UTRs based on Ensembl (v54) annotation; (c) proximal promoters defined by H3K4me3 marks in a human PFC age-series ChIP-Seq experiment, combining all 14 samples together and choosing peaks with posterior possibility ≥0.95 [19]; (d) putative enhancer sites based on the presence of H3K4me1 “broad” peaks, presence of DNAseI hypersensitive sites, and the absence of H3K4me3 “broad” peaks (which is a promoter mark) identified in the Encode project, in an epithelial cell line (Hmec) (http://hgdownload.cse.ucsc.edu/goldenPath/hg18/encodeDCC/) [18] and assigned to nearest human Ensembl gene within 100 kb; (e) putative enhancer sites defined similar to (d) but showing peaks in a minimum of three cell lines among the seven main cell lines used in the Encode project; and (f) putative enhancer sites bound by the transcriptional activator CBP in a mouse brain ChIP-Seq experiment [20]. For estimating species-specific mutations for each of these regions, we extracted the corresponding regions from the human (hg18) genome, projected these onto chimpanzee (panTro2) and rhesus macaque (rheMac2) genomes using “Liftover,” and aligned the corresponding sequences with MUSCLE (v3.7, http://www.drive5.com/muscle). As inflated mutation estimates can arise due to homology or genome alignment problems, we removed the 5% upper fraction of mutation estimates. In Figure 2A–B, the variables (divergence or cell type specificity) were scaled by subtracting the average value across all expressed genes in the tissue, to represent deviation from the transcriptome average. To improve visualization within the same barplot, each variable was also further rescaled by multiplying with a constant.

### Cell Line Transfections

Construct sequences used and the details of the transfection experiment are provided in Text S1. Briefly, we transfected two human neuroblastoma cell lines, SH-SY5Y and SK-N-SH, with three miRNA constructs containing mature sequences for miR-92a, miR-454, and miR-320b, based on miRBase [48], as well as two negative controls. Cells were incubated in DMEM (Hyclone) + 10% FBS (Hyclone) medium and transfected using the Lipofectamine 2000 (Invitrogen) standard protocol (Text S1). Twenty-four hours after transfection, cells were washed twice with PBS and RNA was isolated using Trizol (Invitrogen). Total RNA was labeled and hybridized to Affymetrix HG U133Plus2.0 arrays following standard protocols. Microarray expression data were processed as described above for other datasets. Expression levels for the negative control-transfected cell lines were subtracted from the expression of miRNA-transfected cell line, for each miRNA and cell line type separately, yielding a measure of miRNA effect size. For Figure 5A, we calculated the mean effect size per gene per miRNA across the two cell lines. Treating each cell line separately, as well as together, gave us significant results, and targets verified in each cell line (i.e. TargetScan targets that show inhibition upon transfection) also showed significant overlap (Figure S8).

### In Situ Hybridizations

Hybridizations were performed following [49], and details can be found in Text S1. Briefly, tissue sections were collected on Superfrost/plus slides (Fisher). Tissue was fixed with formaldehyde and subsequently treated with proteinase K [50]. After washing by PBS, sections were acetylated and incubated in humidified bioassay trays for prehybridization at 50°C for 4 h in hybridization buffer [49],[51]. This was followed by an overnight hybridization step using the DIG-labeled LNA oligonucleotide probes complementary to the target miRNAs. Sections were rinsed at 50°C and washed multiple times in SSC buffer. The in situ signal was detected by incubation with alkaline phosphatase (AP)-conjugated anti-DIG antibody, using NBT/BCIP as substrate.

## Supporting Information

Figure S1 Age distributions and variance analysis. (A) Age distribution of subjects used in the gene microarray analysis. Each point represents an individual; technical replicates are shown as additional points below. Only one of the two replicates was used in the main analysis. Vertical arrows indicate 10 individuals per species used to control sample size biases (Text S1). Colors indicate species (red, human; blue, chimpanzee; green, macaque). The *x*-axis represents individuals' age in fourth root (age^1/4^) scale. (B) Sources of total variation in the PFC and CBC microarray datasets, estimated as random effects in a mixed linear model [52],[53]. RIN, RNA integrity number; Batch, the batch information of samples. The graph shows the mean values across all detected genes in a dataset.
(PDF)

Figure S2 Expression-age trajectory correlations among species and comparison to published datasets. (A) Correlation of expression-age trajectories between each pair of species across age-related genes (i.e. genes with significantly varying expression throughout lifespan; Text S1): *n* = 6,234 in PFC and *n* = 5,526 in CBC. The *y*-axis shows the relative frequency of Pearson correlations between interpolated trajectories of each species. (B) Comparison of age-related expression changes in the PFC microarray dataset with two published age-series. We chose 6,234 age-related genes identified in the PFC dataset and all corresponding detected genes in the second dataset: 5,671 genes in a human lifespan comparison [8], 4,131 genes in a chimpanzee lifespan comparison, 1,751 genes in a macaque lifespan comparison, and 5,292 genes in a human aging comparison [54]. (C) Comparison of human-chimpanzee and human-macaque differences across lifespan with a published primate PFC expression age-series [8]. The *y*-axis shows the relative frequency of Pearson correlations between interpolated expression-age trajectories from two datasets, calculated for each commonly expressed gene: 7,271 genes in the human-chimpanzee comparison, and 2,740 genes in the human-macaque comparison. Note that these species differences represent combinations of constitutive and pattern divergence.
(PDF)

Figure S3 Human versus chimpanzee expression divergence. (A–D) Leftmost graphs show the number of genes assigned to different types of divergence in the two brain regions analyzed. The middle and rightmost graphs show distributions of log_2_ human-chimpanzee branch ratios across three divergent gene sets in PFC and CBC, respectively. The results are based on an analysis using (A) the full dataset and chronological age, as presented in the main text; (B) sub-sampling the three gene sets to equalize mean brain expression level distributions (Text S1); (C) choosing the same number of individuals (*n* = 10) with similar age distributions across lifespan for all three species (see Figure S1a); and (D) transforming ages in order to correct for life-history differences (Text S1). For median branch length ratios and significance testing of the distributions' skewness, see Table S3. (E) The numbers of genes showing significant divergence among all species (“unspecific”) or showing significant divergence (*F* test *p*<1-e3) in only one species; e.g. a human-specific gene shows significant difference in both human-chimpanzee and human-macaque comparisons, but not in the chimpanzee-macaque comparison. Note that this result supports the analysis based on expression difference NJ trees, with comparable human-chimpanzee divergence among constitutive genes, and extreme human divergence among type III genes, particularly pronounced in the PFC.
(PDF)

Figure S4 Analysis of the mRNA-sequencing dataset. (A) Principle components of PFC and CBC RNA-seq datasets. Proportion of variance explained by each component is shown in axes labels in parentheses. Each point represents a sample, represented as “h,” human; “c,” chimpanzee; “m,” macaque; “1,” new born; “2,” young adult. In total, 15,183 and 14,941 Ensembl human genes were detected in PFC and CBC datasets, respectively. (B) Proportions of detected genes showing a higher human-macaque expression distance compared to chimpanzee-macaque distance (Text S1), suggesting a trend of higher divergence on the human lineage. The genes are chosen from gene sets identified in the microarray analysis; the gene numbers are shown inside the bars. Error bars indicate 95% bootstrap confidence intervals.
(PDF)

Figure S5 Expression breadth and sequence divergence in CBC. The panels contain the same information as in Figure 2, but it uses gene sets defined using CBC expression instead of PFC expression. (A) Mean expression breadth or nervous system specificity among divergent gene types in CBC, relative to all expressed genes in CBC. Expression breadth indicates the number of cell types in which a gene is expressed (Text S1). Neuron/glia-specificity measures were obtained from Gene Ontology (“GO”) [14] or a mouse experiment (“mouse”) [47]. (B) Median sequence divergence among divergent gene types in the CBC, relative to all expressed genes in CBC. Promoter (±200 bp around the transcription start site) and 3′UTR divergence is estimated from pan-mammalian Phastcons scores [46]; coding divergence reflects human-mouse or human-rhesus macaque dN/dS. Error bars represent 95% bootstrap intervals. In both panels, to control for influence of expression level on conservation or expression breadth, we used gene subsets with equalized mean expression level distributions. The *y*-axis is not to scale among different variables.
(PDF)

Figure S6 Correlations between species-specific divergence in PFC and CBC. We used genes showing the same divergence type in both regions. For each gene, species-specific expression divergence was estimated from the branch lengths on the NJ tree. The trees were constructed using Euclidean distances between a pair of species' expression-age trajectories. Spearman correlation coefficients (rho) and *p* values are indicated below the panels.
(PDF)

Figure S7 CBC miRNA transcriptome PCA and quality control. (A) Principle component analysis results for the CBC miRNA transcriptome (207 detected miRNAs). Each point represents a sample, with its age in days or years (“1d” denoting 1 day, “12” denoting 12 years). Colors represent species (red, human; blue, chimpanzee; green, macaque). (B) Correlation between 167 age-related expression profiles or human-macaque differences between miRNA microarray and RNA-sequencing datasets in the PFC, using a published PFC miRNA-seq dataset [44]. The *y*-axis shows the relative frequency of Pearson correlations between interpolated expression-age trajectories from the two platforms, calculated for each commonly detected miRNA.
(PDF)

Figure S8 Gene expression shifts in miRNA-transfected cell lines. (A) The colored and grey lines show the cumulative density (*y*-axis) of the expression change magnitude upon transfection (*x*-axis), for target genes predicted by TargetScan among PFC type III genes, and all other type III genes, respectively. The *x*-axis is calculated as the expression level difference between miRNA-transfected and negative control-transfected neuroblastoma cell lines, per gene (Materials and Methods). The *p* values were calculated by one-sided Wilcoxon tests. Columns on the right and left show results for SK-N-SH and SH-SY5Y, respectively. In comparison, Figure 4B shows the mean expression change across the two cell lines. (B) Overlap between PFC type III genes showing down-regulation upon transfection in the two cell lines. The *p* values are based on one-sided hypergeometric tests and use genes showing up-regulation as background.
(PDF)

Figure S9 Sequence conservation and sequence-expression divergence correlations. The same analysis was done as in Figure 2B and 2D, but using definitions of regulatory regions not based on Ensembl annotation. (A) Average divergence per PFC gene set using different measures, normalized to average of all expressed genes. (B) Spearman correlation coefficient between pan-mammalian sequence divergence and type III gene expression divergence on each lineage. Promoter (neuron): proximal promoter based on regions identified by H3K4me3 marks in human PFC [19]; Enhancer (Hmec): putative enhancer sites based on the chromatin modification marks (presence of H3K4me1 and DNAseI hypersensitive sites and absence of H3K4me3 sites identified by the ENCODE project in an epithelial cell line (Hmec) [18]); Enhancer (Encode-union): putative enhancer sites defined as Hmec, but showing peaks in minimum three ENCODE cell lines; Enhancer (mouse): putative enhancer sites bound by the transcriptional activator CBP in a mouse brain ChIP-Seq experiment [20].
(PDF)

Table S1 Sample information. PMI, post-mortem interval in hours; RIN, RNA integrity number (Agilent 2100 Bioanalyzer system); RNA array, Affymetrix Human Gene 1.0 ST array; miRNA array, Agilent Human MicroRNA Microarray (G4471A, Agilent Technologies); RNA-seq, mRNA profiling using Illumina Genome Analyzer II; PFC, dorsolateral prefrontal cortex; CBC, cerebellar cortex. The “Experiments” column indicates in which experiments an individual's tissue samples were used. a, RNA microarray for PFC; b, RNA microarray for CBC; e, RNA-seq for PFC (pool); f, RNA-seq for CBC (pool); h, miRNA microarray for PFC; I, miRNA microarray for CBC. In the columns containing microarray information, b1 and b2 indicate which individuals were included in the 1st and 2nd experimental batches for that experiment, respectively. In the columns containing RNA-seq information, “newborn,” “young,” and “old” indicate of which age pool an individual sample was a part: newborns, young adults, or old adults. ASCVD, arteriosclerotic cardiovascular disease; HASCVD, hypertensive arteriosclerotic cardiovascular disease.
(XLS)

Table S2 Numbers of differentially and non-differentially expressed genes. Genes were sorted into categories based on three tests: (a) age-test, applied per species; (b) differential-expression test, applied for each species comparison; and (c) differential-expression test using standardized data (removing constitutive expression differences among species), applied for each species comparison (Text S1). All *p* values are based on the *F* test. “No difference” indicates genes with no evidence for divergence.
(XLS)

Table S3 Comparison of human versus chimpanzee divergence. Median human-chimpanzee branch length ratios across different divergent gene sets. Positive values indicate longer human branches, and negative values indicate longer chimpanzee branches. We tested log_2_ transformed branch length ratio distributions for median = 0 using the Wilcoxon test. The upper and lower bounds of the 95% confidence interval for the median were estimated by bootstrapping genes within each set 1,000 times. The test was applied under various conditions: using all samples, the full gene sets, chronological ages (“full”); using gene sets equalized for mean brain expression level differences (“equalized expression”); after correcting for life-history differences among species (“normalized life-history”); and using the same number of individuals per species (“same number of indv.”). The first two rows show results for all expressed; in this case, both constitutive and pattern differences contribute to species divergence.
(XLS)

Table S4 Functional characteristics of divergent gene types. Kyoto Encyclopedia of Genes and Genomes (KEGG) and Gene Ontology biological process (GO) categories in which a divergent gene set shows enrichment, compared to the other categories (the background). “Test” and “Other” indicate the number of genes in a functional category belonging to the tested gene set or the background, respectively. HT, one-sided hypergeometric test.
(XLS)

Table S5 Expression divergence-sequence divergence correlations in PFC. Spec., species-specific divergence represents the number of mutations assigned to one of three species in a regulatory region; Pan-mam., pan-mammalian divergence, calculated as the negative Phastcons score; Prom, proximal promoter based on Ensembl; Prom2, proximal promoter based on regions identified by H3K4me3 marks in human PFC [19]; 3utr, 3′UTR based on Ensembl; Enhmec and Enhuni, putative enhancer based on the chromatin modification marks identified by the Encode project in Hmec or multiple cell lines, respectively [18]; Enhmus, putative enhancer based CBP ChIP-Seq experiments in the mouse brain [20]. See Materials and Methods for details. Significant correlations are marked bold. No correction for multiple testing was performed. Brown marked rows show correlation between type III divergence in PFC and pan-mammalian sequence divergence.
(XLS)

Table S6 Excess of predicted regulator-target correlations. Correlations between regulator versus predicted target pairs' expression profiles were compared to correlations between regulator versus non-target pairs (genes that are predicted to be targeted by other regulators), using the hypergeometric test (HT). We tested excess of negative and positive correlations for miRNA-target and TF-target pairs, respectively. We used two correlation cutoffs, and also two types of correlation: (1) is based on raw expression profiles and reflects both age and species effects, while (2) is based on expression differences among species (Text S1).
(XLS)

Table S7 Micro RNAs with target enrichment among PFC type III genes. Micro RNAs showing predicted target enrichment among PFC type III genes, compared to the other divergent gene types (the background). In total 596 miRNAs in the TargetScan table were tested (Text S1) and only those passing the *p*<0.05 cutoff are shown. “Test” and “Other” indicate the number of genes in a functional category belonging to the tested gene set or the background, respectively. HT, one-sided hypergeometric test; FDR, false discovery rate estimated from 1,000 permutations, indicating the average number of genes expected to appear significant at a *p* value cutoff by chance.
(XLS)

Table S8 Candidate miRNA regulators of PFC developmental remodeling. Micro RNAs and their targets predicted by (i) enrichment of a miRNA targets among PFC type III genes, compared to other divergent gene types (HT *p*<0.05), (ii) excess of negative correlations between the miRNA's expression profile and its type III targets' expression profiles, compared to negative correlations (*r*<−0.75) between miRNAs that are not enriched among type III genes and their type III targets (one-sided binomial test *p*<0.05). We used two kinds of correlation, one based on raw expression profiles, and the other based on expression differences between pairs of species. See Text S1 for details.
(XLS)

Table S9 Functional characteristics of verified miRNA targets. Kyoto Encyclopedia of Genes and Genomes (KEGG) categories in which verified targets of an miRNA show enrichment, compared to the other categories (the background). “Test” and “Other” indicate the number of genes in a functional category belonging to the tested gene set or the background, respectively. HT, one-sided hypergeometric test. Verified targets are defined using TargetScan, negative correlations in the time series, and inhibition by miRNA-transfection in cell lines. miR_320b targets do not show significant enrichment. Only miR-454 targets show significant enrichment using GO annotation (unpublished data). “Union” indicates the union of verified targets for the miR-320b, miR-454, and miR-92a.
(XLS)

Text S1 Supporting methods.
(DOC)
