# Supplementary figures and images for: MicroRNA-Driven Developmental Remodeling in the Brain Distinguishes Humans from Other Primates
Source: PLoS Biol. 2011 Dec 6;9(12):e1001214. doi: 10.1371/journal.pbio.1001214 (PMC3232219; doi:10.1371/journal.pbio.1001214)

**A**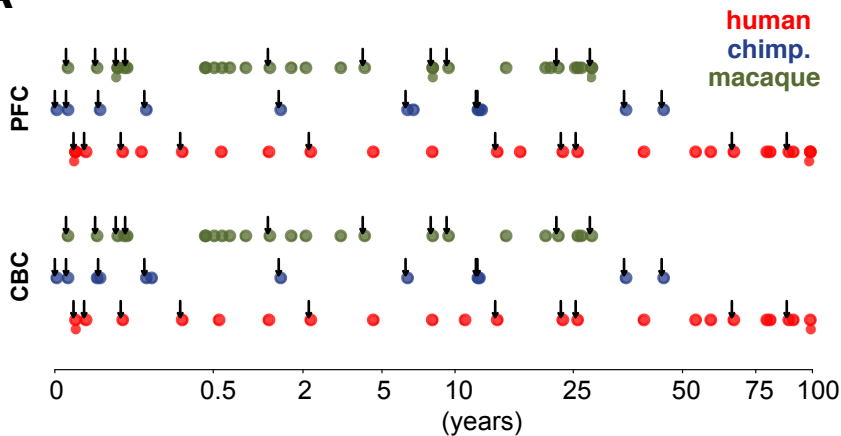**B**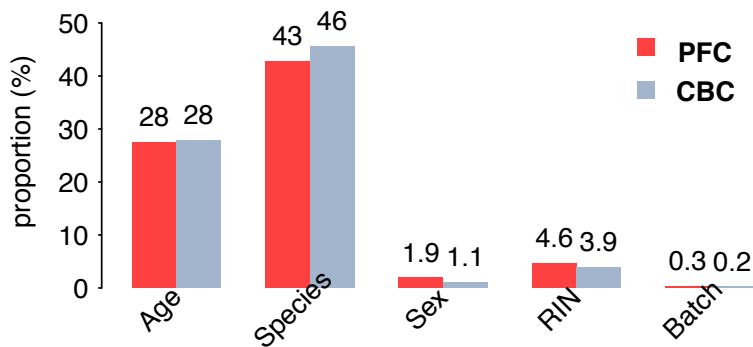

Supplement: Figure S1 — Age distributions and variance analysis. (A) Age distribution of subjects used in the gene microarray analysis. Each point represents an individual; technical replicates are shown as additional points below. Only one of the two replicates was used in the main analysis. Vertical arrows indicate 10 individuals per species used to control sample size biases (Text S1). Colors indicate species (red, human; blue, chimpanzee; green, macaque). The x-axis represents individuals' age in fourth root (age1/4) scale. (B) Sources of total variation in the PFC and CBC microarray datasets, estimated as random effects in a mixed linear model [52],[53]. RIN, RNA integrity number; Batch, the batch information of samples. The graph shows the mean values across all detected genes in a dataset. (PDF) [file pbio.1001214.s001.pdf]

**A****PFC**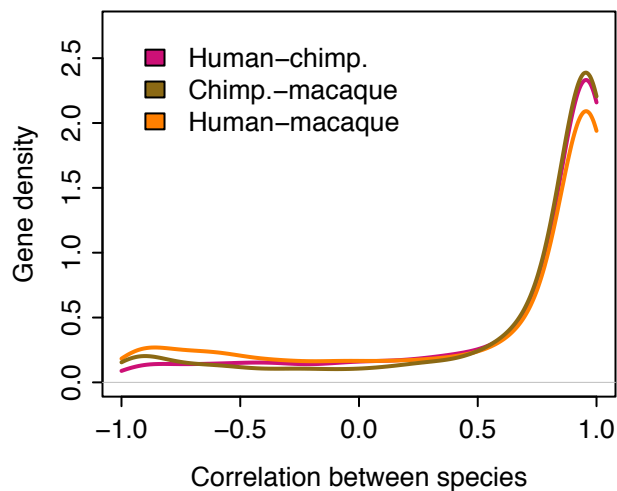**CBC**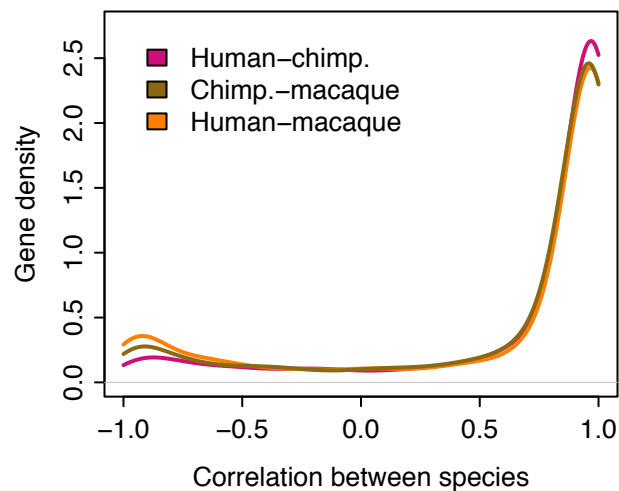**B**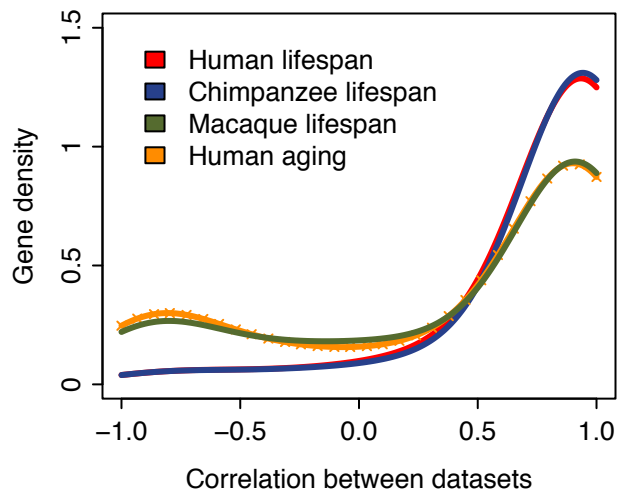**C**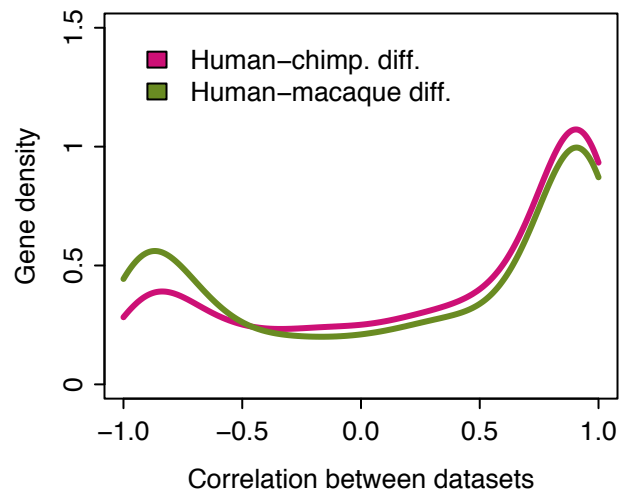

Supplement: Figure S2 — Expression-age trajectory correlations among species and comparison to published datasets. (A) Correlation of expression-age trajectories between each pair of species across age-related genes (i.e. genes with significantly varying expression throughout lifespan; Text S1): n = 6,234 in PFC and n = 5,526 in CBC. The y-axis shows the relative frequency of Pearson correlations between interpolated trajectories of each species. (B) Comparison of age-related expression changes in the PFC microarray dataset with two published age-series. We chose 6,234 age-related genes identified in the PFC dataset and all corresponding detected genes in the second dataset: 5,671 genes in a human lifespan comparison [8], 4,131 genes in a chimpanzee lifespan comparison, 1,751 genes in a macaque lifespan comparison, and 5,292 genes in a human aging comparison [54]. (C) Comparison of human-chimpanzee and human-macaque differences across lifespan with a published primate PFC expression age-series [8]. The y-axis shows the relative frequency of Pearson correlations between interpolated expression-age trajectories from two datasets, calculated for each commonly expressed gene: 7,271 genes in the human-chimpanzee comparison, and 2,740 genes in the human-macaque comparison. Note that these species differences represent combinations of constitutive and pattern divergence. (PDF) [file pbio.1001214.s002.pdf]

**A**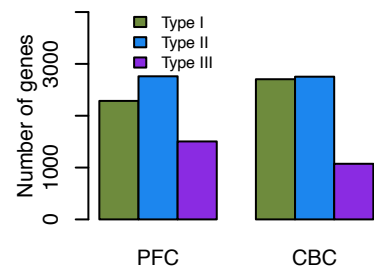**PFC**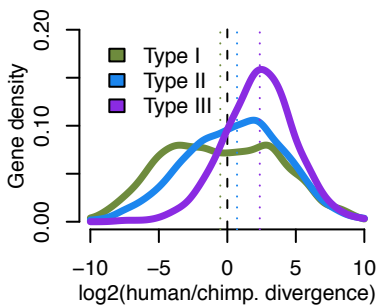**CBC**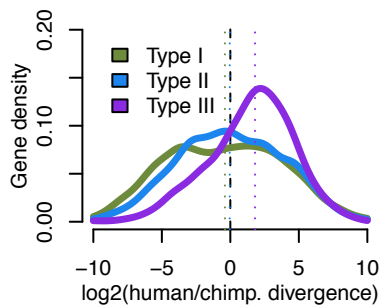**B**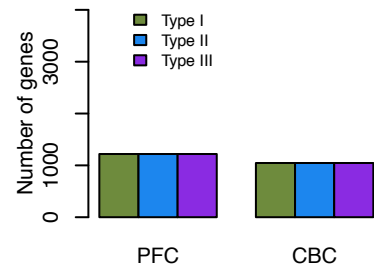**PFC**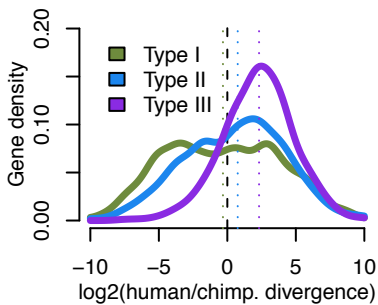**CBC**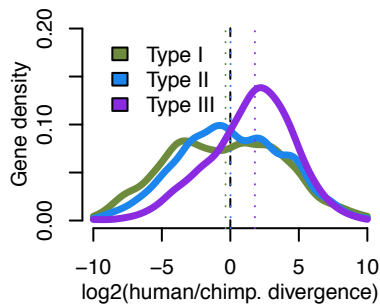**C**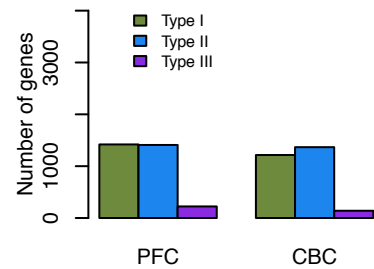**PFC**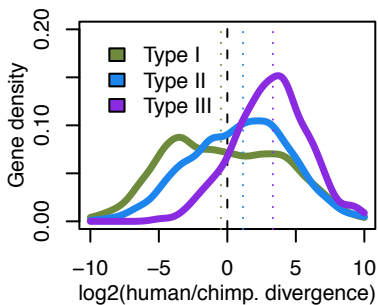**CBC**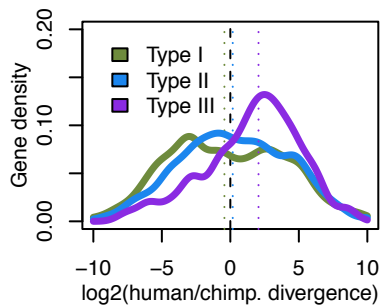**D**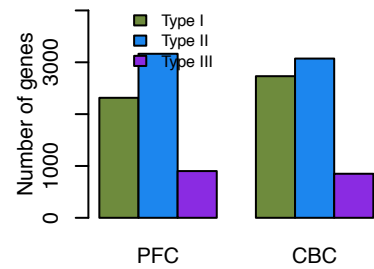**PFC**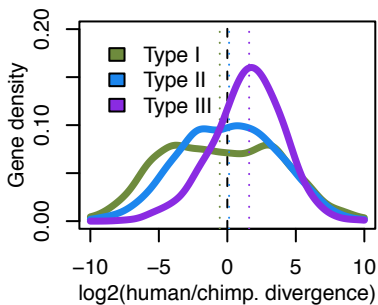**CBC**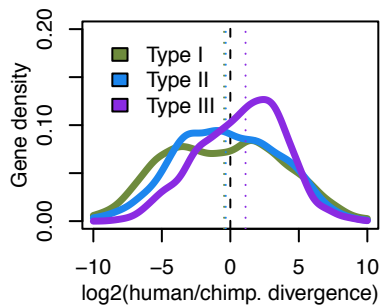**E**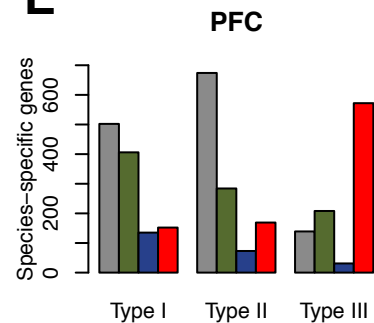**PFC**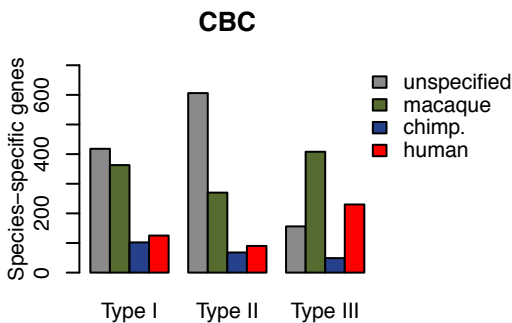**CBC**

Supplement: Figure S3 — Human versus chimpanzee expression divergence. (A–D) Leftmost graphs show the number of genes assigned to different types of divergence in the two brain regions analyzed. The middle and rightmost graphs show distributions of log2 human-chimpanzee branch ratios across three divergent gene sets in PFC and CBC, respectively. The results are based on an analysis using (A) the full dataset and chronological age, as presented in the main text; (B) sub-sampling the three gene sets to equalize mean brain expression level distributions (Text S1); (C) choosing the same number of individuals (n = 10) with similar age distributions across lifespan for all three species (see Figure S1a); and (D) transforming ages in order to correct for life-history differences (Text S1). For median branch length ratios and significance testing of the distributions' skewness, see Table S3. (E) The numbers of genes showing significant divergence among all species (“unspecific”) or showing significant divergence (F test p<1-e3) in only one species; e.g. a human-specific gene shows significant difference in both human-chimpanzee and human-macaque comparisons, but not in the chimpanzee-macaque comparison. Note that this result supports the analysis based on expression difference NJ trees, with comparable human-chimpanzee divergence among constitutive genes, and extreme human divergence among type III genes, particularly pronounced in the PFC. (PDF) [file pbio.1001214.s003.pdf]

**A**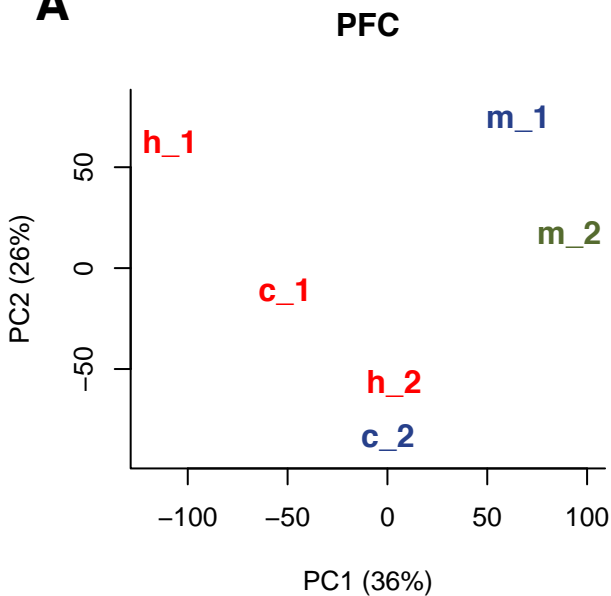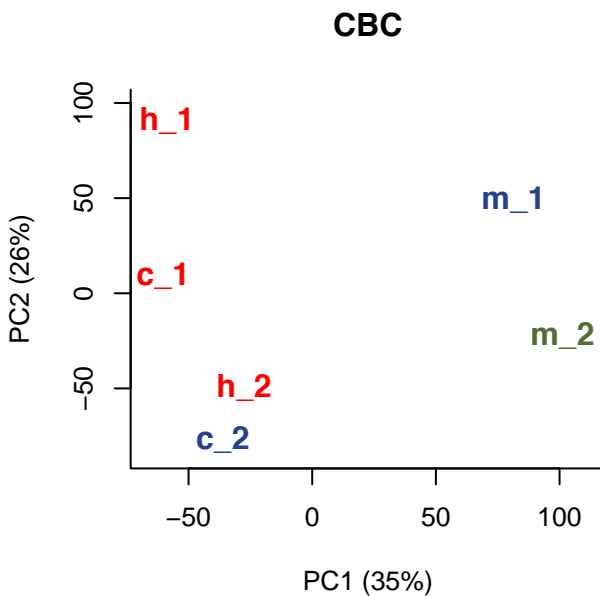**B**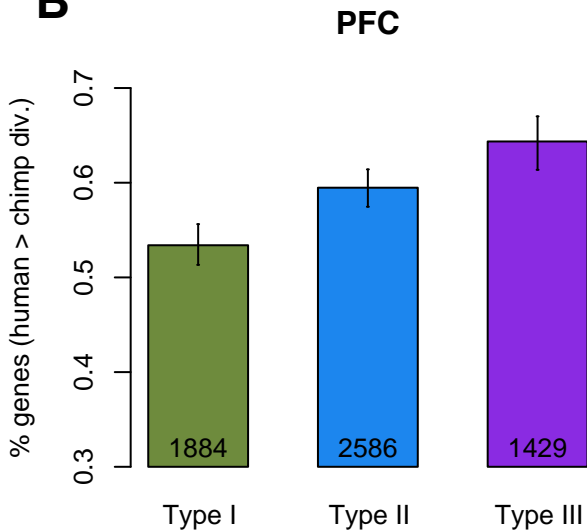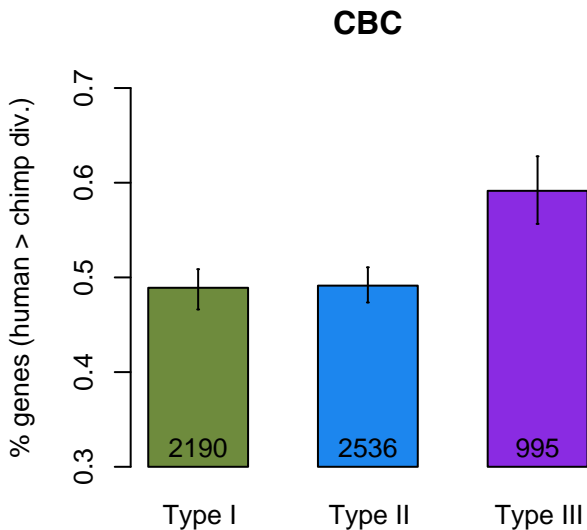

Supplement: Figure S4 — Analysis of the mRNA-sequencing dataset. (A) Principle components of PFC and CBC RNA-seq datasets. Proportion of variance explained by each component is shown in axes labels in parentheses. Each point represents a sample, represented as “h,” human; “c,” chimpanzee; “m,” macaque; “1,” new born; “2,” young adult. In total, 15,183 and 14,941 Ensembl human genes were detected in PFC and CBC datasets, respectively. (B) Proportions of detected genes showing a higher human-macaque expression distance compared to chimpanzee-macaque distance (Text S1), suggesting a trend of higher divergence on the human lineage. The genes are chosen from gene sets identified in the microarray analysis; the gene numbers are shown inside the bars. Error bars indicate 95% bootstrap confidence intervals. (PDF) [file pbio.1001214.s004.pdf]

**A**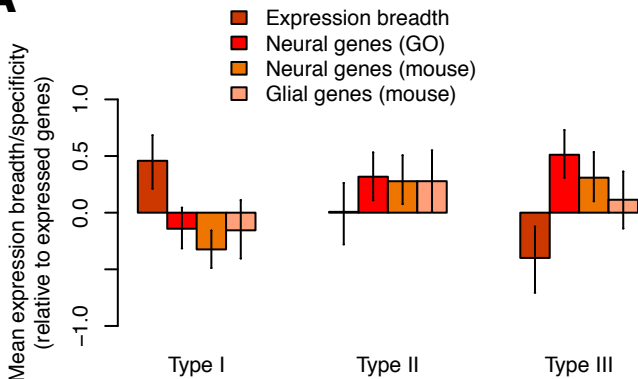**B**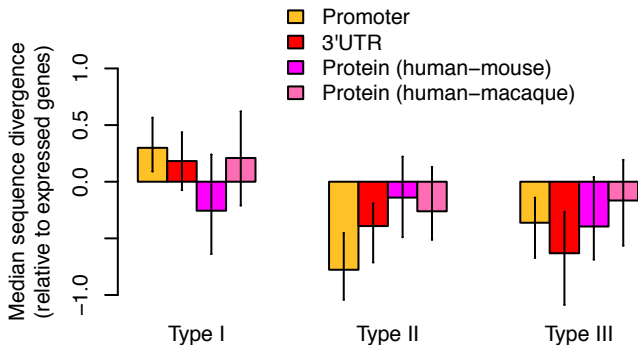

Supplement: Figure S5 — Expression breadth and sequence divergence in CBC. The panels contain the same information as in Figure 2, but it uses gene sets defined using CBC expression instead of PFC expression. (A) Mean expression breadth or nervous system specificity among divergent gene types in CBC, relative to all expressed genes in CBC. Expression breadth indicates the number of cell types in which a gene is expressed (Text S1). Neuron/glia-specificity measures were obtained from Gene Ontology (“GO”) [14] or a mouse experiment (“mouse”) [47]. (B) Median sequence divergence among divergent gene types in the CBC, relative to all expressed genes in CBC. Promoter (±200 bp around the transcription start site) and 3′UTR divergence is estimated from pan-mammalian Phastcons scores [46]; coding divergence reflects human-mouse or human-rhesus macaque dN/dS. Error bars represent 95% bootstrap intervals. In both panels, to control for influence of expression level on conservation or expression breadth, we used gene subsets with equalized mean expression level distributions. The y-axis is not to scale among different variables. (PDF) [file pbio.1001214.s005.pdf]

**A**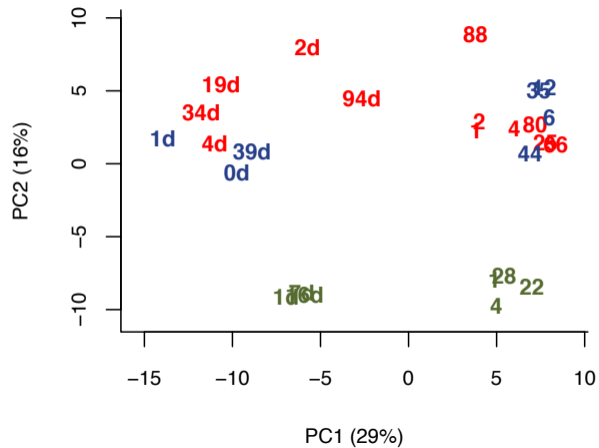**B**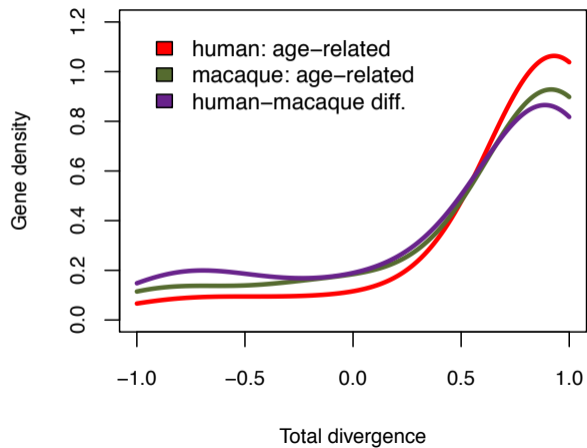

Supplement: Figure S7 — CBC miRNA transcriptome PCA and quality control. (A) Principle component analysis results for the CBC miRNA transcriptome (207 detected miRNAs). Each point represents a sample, with its age in days or years (“1d” denoting 1 day, “12” denoting 12 years). Colors represent species (red, human; blue, chimpanzee; green, macaque). (B) Correlation between 167 age-related expression profiles or human-macaque differences between miRNA microarray and RNA-sequencing datasets in the PFC, using a published PFC miRNA-seq dataset [44]. The y-axis shows the relative frequency of Pearson correlations between interpolated expression-age trajectories from the two platforms, calculated for each commonly detected miRNA. (PDF) [file pbio.1001214.s007.pdf]

**A**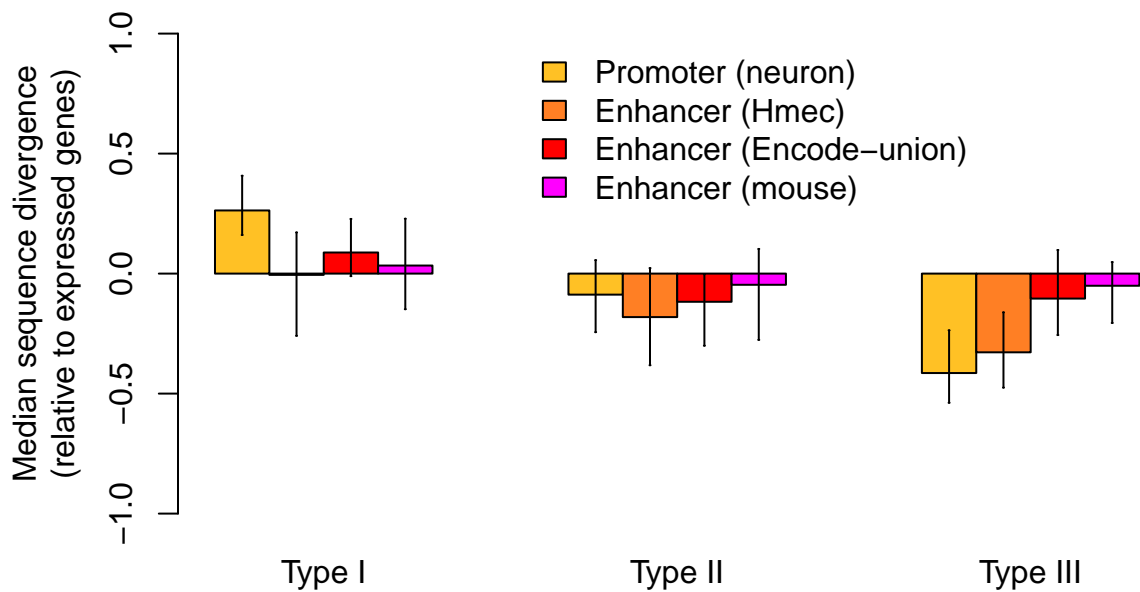**B**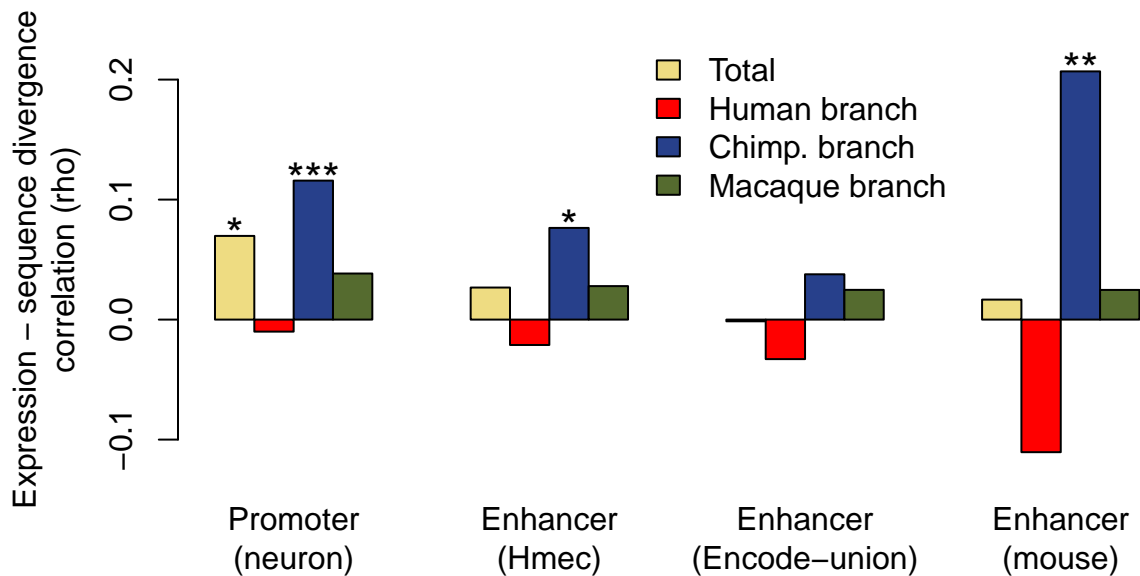

Supplement: Figure S9 — Sequence conservation and sequence-expression divergence correlations. The same analysis was done as in Figure 2B and 2D, but using definitions of regulatory regions not based on Ensembl annotation. (A) Average divergence per PFC gene set using different measures, normalized to average of all expressed genes. (B) Spearman correlation coefficient between pan-mammalian sequence divergence and type III gene expression divergence on each lineage. Promoter (neuron): proximal promoter based on regions identified by H3K4me3 marks in human PFC [19]; Enhancer (Hmec): putative enhancer sites based on the chromatin modification marks (presence of H3K4me1 and DNAseI hypersensitive sites and absence of H3K4me3 sites identified by the ENCODE project in an epithelial cell line (Hmec) [18]); Enhancer (Encode-union): putative enhancer sites defined as Hmec, but showing peaks in minimum three ENCODE cell lines; Enhancer (mouse): putative enhancer sites bound by the transcriptional activator CBP in a mouse brain ChIP-Seq experiment [20]. (PDF) [file pbio.1001214.s009.pdf]
