# Supplementary material for: MicroRNA-Driven Developmental Remodeling in the Brain Distinguishes Humans from Other Primates
Source: PLoS Biol. 2011 Dec 6;9(12):e1001214. doi: 10.1371/journal.pbio.1001214 (PMC3232219; doi:10.1371/journal.pbio.1001214)
Supplement: Figure S6 — Correlations between species-specific divergence in PFC and CBC. We used genes showing the same divergence type in both regions. For each gene, species-specific expression divergence was estimated from the branch lengths on the NJ tree. The trees were constructed using Euclidean distances between a pair of species' expression-age trajectories. Spearman correlation coefficients (rho) and p values are indicated below the panels. (PDF) [file pbio.1001214.s006.pdf]

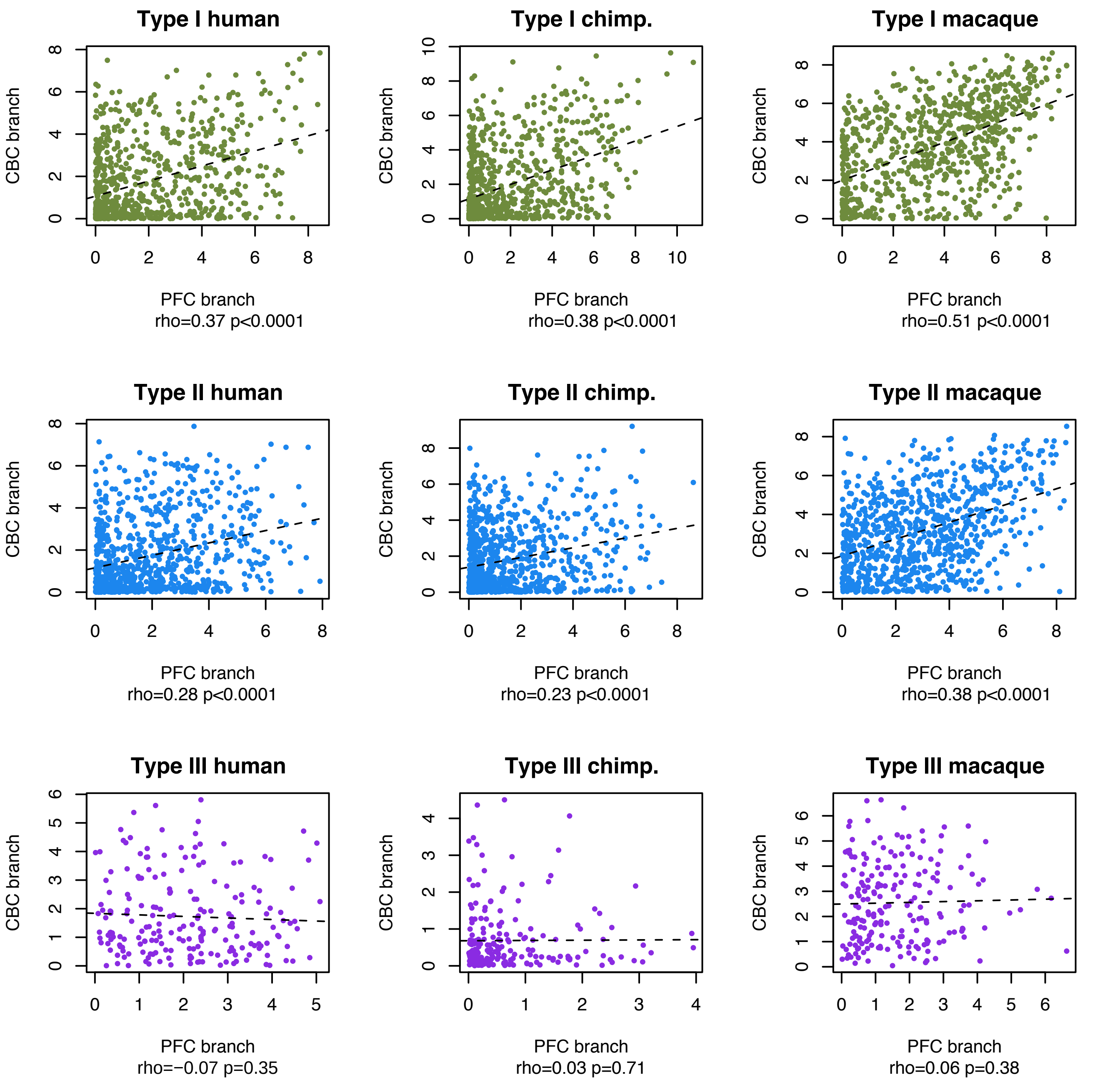
Type I human
Type I chimp.
Type I macaque
Type II human
Type II chimp.
Type II macaque
Type III human
Type III chimp.
Type III macaque
CBC branch
PFC branch
rho=0.37 p<0.0001
rho=0.38 p<0.0001
rho=0.51 p<0.0001
rho=0.28 p<0.0001
rho=0.23 p<0.0001
rho=0.38 p<0.0001
rho=−0.07 p=0.35
rho=0.03 p=0.71
rho=0.06 p=0.38
