# Supplementary material for: MicroRNA-Driven Developmental Remodeling in the Brain Distinguishes Humans from Other Primates
Source: PLoS Biol. 2011 Dec 6;9(12):e1001214. doi: 10.1371/journal.pbio.1001214 (PMC3232219; doi:10.1371/journal.pbio.1001214)
Supplement: Figure S8 — Gene expression shifts in miRNA-transfected cell lines. (A) The colored and grey lines show the cumulative density (y-axis) of the expression change magnitude upon transfection (x-axis), for target genes predicted by TargetScan among PFC type III genes, and all other type III genes, respectively. The x-axis is calculated as the expression level difference between miRNA-transfected and negative control-transfected neuroblastoma cell lines, per gene (Materials and Methods). The p values were calculated by one-sided Wilcoxon tests. Columns on the right and left show results for SK-N-SH and SH-SY5Y, respectively. In comparison, Figure 4B shows the mean expression change across the two cell lines. (B) Overlap between PFC type III genes showing down-regulation upon transfection in the two cell lines. The p values are based on one-sided hypergeometric tests and use genes showing up-regulation as background. (PDF) [file pbio.1001214.s008.pdf]

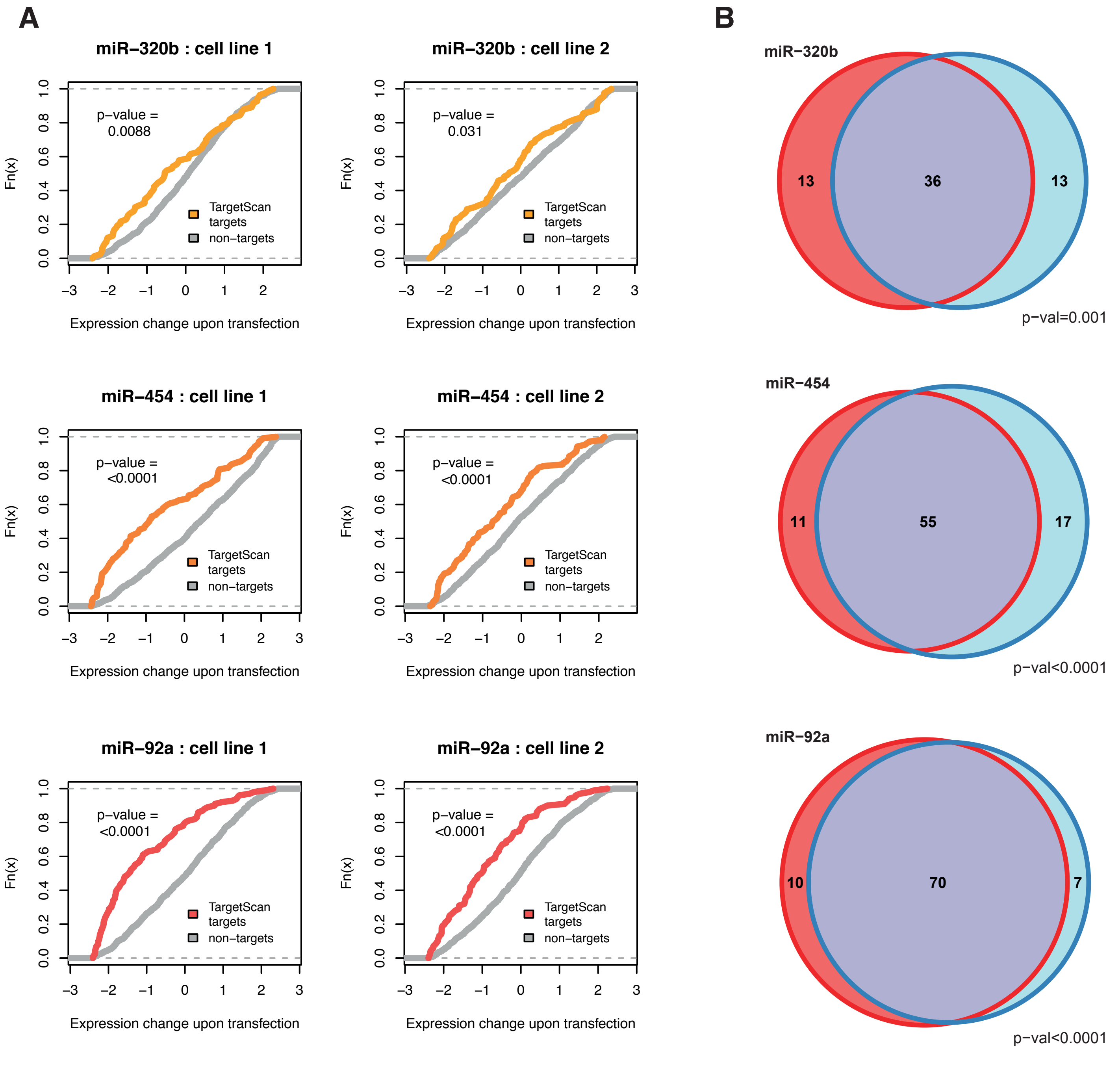
A
miR−320b : cell line 1
p−value = 0.0088
TargetScan targets
non−targets
Fn(x)
Expression change upon transfection
miR−320b : cell line 2
p−value = 0.031
miR−454 : cell line 1
p−value = <0.0001
miR−454 : cell line 2
p−value = <0.0001
miR−92a : cell line 1
p−value = <0.0001
miR−92a : cell line 2
p−value = <0.0001
B
miR−320b
13
36
13
p−val=0.001
miR−454
11
55
17
p−val<0.0001
miR−92a
10
70
7
p−val<0.0001
