# Supplementary material for: MicroRNA-Driven Developmental Remodeling in the Brain Distinguishes Humans from Other Primates
Source: PLoS Biol. 2011 Dec 6;9(12):e1001214. doi: 10.1371/journal.pbio.1001214 (PMC3232219; doi:10.1371/journal.pbio.1001214)
Supplement: Text S1 — Supporting methods. (DOC) [file pbio.1001214.s019.doc]

**MicroRNA-driven developmental remodeling in the brain distinguishes humans from other primates**

Supporting Methods

Sample collection. [2](#__RefHeading___Toc180957276)

RNA microarray hybridization. [2](#__RefHeading___Toc180957277)

Microarray data preprocessing. [3](#__RefHeading___Toc180957278)

The standardized dataset - constitutive differences among species removed. [3](#__RefHeading___Toc180957279)

Age scale. [4](#__RefHeading___Toc180957280)

Microarray data quality assessment and variance estimation. [4](#__RefHeading___Toc180957281)

Interpolation of expression-age trajectories. [5](#__RefHeading___Toc180957282)

Comparison to published datasets. [5](#__RefHeading___Toc180957283)

Principle components analysis. [5](#__RefHeading___Toc180957284)

Age-test. [6](#__RefHeading___Toc180957285)

Differential expression test. [6](#__RefHeading___Toc180957286)

Testing for pattern differences. [7](#__RefHeading___Toc180957287)

Definition of three divergence types. [8](#__RefHeading___Toc180957288)

Estimating expression divergence. [8](#__RefHeading___Toc180957289)

Equalizing mean expression among gene sets. [9](#__RefHeading___Toc180957290)

Identifying species-specific genes. [9](#__RefHeading___Toc180957291)

Normalizing life-history among species. [10](#__RefHeading___Toc180957292)

RNA sequencing. [10](#__RefHeading___Toc180957293)

RNA-sequencing analysis. [11](#__RefHeading___Toc180957294)

Sequence divergence estimates. [12](#__RefHeading___Toc180957295)

Identification of species-specific mutations in regulatory regions. [12](#__RefHeading___Toc180957296)

Functional enrichment analysis. [13](#__RefHeading___Toc180957297)

Expression breadth and cell type-specificity analyses. [13](#__RefHeading___Toc180957298)

MicroRNA and TF target identification. [14](#__RefHeading___Toc180957299)

MicroRNA microarrays. [15](#__RefHeading___Toc180957300)

Regulator-target correlations. [16](#__RefHeading___Toc180957301)

Identification of candidate regulators. [17](#__RefHeading___Toc180957302)

Verified miRNA target lists and functional enrichment tests. [18](#__RefHeading___Toc180957303)

Analysis of mouse developmental series. [18](#__RefHeading___Toc180957304)

Cell line transfections. [19](#__RefHeading___Toc180957305)

*In situ* hybridizations. [20](#__RefHeading___Toc180957306)

Supporting References [21](#__RefHeading___Toc180957307)

# Sample collection.

We used prefrontal cortex (PFC) and cerebellar cortex (CBC) samples from postmortem brains of 33 human (aged 0-98 years), 14 chimpanzee (aged 0-44 years), and 34 rhesus macaque individuals (aged 0-28 years) (Table S1). Human samples were obtained from the NICHD Brain and Tissue Bank for Developmental Disorders at the University of Maryland, USA, the Netherlands Brain Bank, Amsterdam, Netherlands, and the Chinese Brain Bank Center, Wuhan, China. Informed consent for use of human tissues for research was obtained in writing from all donors or their next of kin. All subjects were defined as normal by forensic pathologists at the corresponding brain bank. All subjects suffered sudden death with no prolonged agonal state. Chimpanzee samples were obtained from the Yerkes Primate Center, GA, USA, the Anthropological Institute & Museum of the University of Zürich-Irchel, Switzerland, and the Biomedical Primate Research Centre, Netherlands. Rhesus macaque samples were obtained from the Suzhou Experimental Animal Center, China. All non-human primates used in this study suffered sudden deaths for reasons other than their participation in this study and without any relation to the tissue used. CBC dissections were made from the cerebellar cortex. PFC dissections were made from the frontal part of the superior frontal gyrus. All samples contained an approximately 2:1 grey matter to white matter volume ratio.

# RNA microarray hybridization.

RNA isolation, hybridization to microarrays, and data preprocessing were performed as described previously (Khaitovich et al. 2005). In brief, total RNA was extracted from 100 mg of dissected CBC and PFC samples (humans, n=22/23; chimpanzees, n=12/12; macaques, n=24/26, respectively; see Table S1) using Trizol (Invitrogen), and purified with the QIAGEN® RNeasy MiniElute kit. RNA quality was assessed with the Agilent® 2100 Bioanalyzer system (Table S1). For each sample, 2 micrograms of isolated RNA was hybridized to an Affymetrix® Human Gene 1.0 ST array. For human and rhesus macaque samples, the microarray experiments were carried out in two batches, with similar age distributions of subjects across batches (Table S1); in total, the hybridizations were performed in five batches per brain region: human1, chimpanzee, rhesus1, human2, rhesus2. Gene expression in two individuals in human PFC and CBC sets, and three individuals in the macaque PFC set, were measured as technical replicates (Table S1).

# Microarray data preprocessing.

All Affymetrix microarray probes were aligned to the reference human, chimpanzee, and rhesus genomes (hg18, panTro2, and rheMac2, respectively) downloaded from the UCSC Genome Browser database, using BLAT (Karolchik et al. 2008). Only probes perfectly and uniquely matching all three genomes were included in the analysis. Probe intensities were exacted using the R package “affy” (Gautier et al. 2004). Probe intensities were corrected for background, log_2_ transformed, and quantile normalized. Here we assume a limited amount of expression differences between species, which is consistent with the general conservation of brain gene expression among primate species (Khaitovich et al. 2005). For each human Ensembl transcript represented on the array (v54, http://www.ensembl.org) (Hubbard et al. 2007), we calculated intensity values using median polishing (Gautier et al. 2004). We then chose one Ensembl gene per transcript; if a gene corresponded to multiple transcripts, we chose the one with highest mean expression level. All primary data is deposited in the NCBI Gene Expression Omnibus database (GEO; <http://www.ncbi.nlm.nih.gov/geo/>) under the accession numbers GSE22570. In order to remove possible batch effects, which can arise due to array hybridizations at different times, we normalized expression levels of each gene to the same mean and standard deviation (separately for each species and brain region). For the two macaque batches, the normalization was based on individuals with overlapping ages, as the age distribution of the two batches were slightly different. In cases where gene expression was measured twice in the same individual as a technical replicate, we randomly chose and removed one of the replicates in the downstream analyses.

# The standardized dataset - constitutive differences among species removed.

To compare age-expression profiles across species while ignoring any constitutive differences, we used the following normalization procedure, which took into account differences in the sample age distributions among species: (i) We chose sample subsets consisting of 10 individuals within each species, with each subset having similar age distribution across the lifespan of each species (using 40, 60 and 120 years as maximum lifespan for macaques, chimpanzees and humans, respectively (http://genomics.senescence.info/species/) (de Magalhães and Costa 2009); (Fig. S1a). (ii) We standardized the mean and standard deviation across the three species based on these subsets: for each gene from each species, we subtracted the 10 individual subset’s mean from all individuals’ expression levels, and divided the result by the 10 individual subset’s standard deviation. We will refer to these as “standardized datasets.”

# Age scale.

For all analyses we used a log_2_ transformed age scale: *i.e.* log_2_(days of age+1), as one chimpanzee is 0 days old. Log-transformation of age is a common procedure to ensure efficient and accurate modeling of developmental changes where the rate of change in the dependent variable, expression level, decreases with increasing age (Clancy et al. 2001; Lu et al. 2001; Shupe et al. 2006). This transformation also yields a relatively uniform error distribution across samples, which is critical for the analysis of variance models used (Sokal and Rohlf 1995).

# Microarray data quality assessment and variance estimation.

We first assessed technical variance in our data using the technical replicates included in the experiment. In both the PFC and the CBC datasets, the pairs of technical replicates showed significantly stronger correlation with each other (across expressed genes) than with other individuals, indicating limited technical variance among samples (one sided t-test, *p*<0.001). We then tested for effects of age, species, sex, and two technical variables, RNA integrity number (RIN, measured by Agilent® 2100 Bioanalyzer) and experimental batch, on expression levels in the PFC and CBC datasets. For each gene, the expression level was used as a response variable to fit a mixed linear model with different sources of variation (age, species, sex, RIN, batch effect) as random effects. We used the “lmer” function with restricted maximum likelihood (REML) estimation in the R package “lme4” (Boedigheimer et al. 2008; Li et al. 2009). Fig. S1b represents the mean of these proportions for each factor, based on all detected genes in each brain region. Postmortem interval (PMI) data was available for humans only and was not included in the model. However, testing the effect of PMI on expression variation using linear models revealed ~5% variance explained by this factor, which was non-significant compared to random permutations (p>0.7).

# Interpolation of expression-age trajectories.

For each gene from each species, we interpolated expression values at 20 equally distributed points along the species’ age range in log2 scale, from the youngest to the oldest individual. When comparing species, we used the three species’ common age-range. We used cubic spline regression for interpolation, restricting the fit to three degrees of freedom, in order to avoid over-fitting. This was applied to the PFC and CBC datasets independently.

# Comparison to published datasets.

We found markedly good correlation between the age-related expression profiles identified in the PFC dataset and the same genes’ expression profiles in published microarray age-series of human, chimpanzee and rhesus macaque dorsolateral PFC expression in development and adulthood (Somel et al. 2009), as well as human PFC aging (Maycox et al. 2009) (Affymetrix® HGU133p2 arrays) (Figure S2). For this, we used 6,234 age-related genes in the PFC dataset (F-test p<0.001 in at least one species). For each dataset, for each species, and for each gene, we interpolated age-expression trajectories at 20 uniformly distributed points across the species’ age-range (the common range between the compared age-series, in log2 scale). We calculated correlations based on these interpolated points. Similarly we compared human-chimpanzee and human-macaque differences across lifespan, using 20 interpolated points across the common range of the four age-series (two species X two datasets) per gene.

# Principle components analysis.

Principle components analysis of the PFC and CBC expression data was performed by singular value decomposition, using the “prcomp” function in the R “stats” package and the “scaling” option to equally weight genes. Fig. 1a was plotted using the R “scatterplot3d” package.

# Age-test.

To choose age-related genes, we tested the effect of age on expression levels using polynomial regression models, as described previously (Somel et al. 2009). Briefly, for each gene, we choose the best polynomial regression with age as a predictor and expression level as a response, using families of polynomial regression models and the “adjusted *r*^2^” criterion (Faraway 2002). The most complex of these models is defined in the formula: Y_ij_ = β_0i_ + β_1i_ A_j_ + β_2i_ A_j_^2^ + β_3i_ A_j_^3^+ ε_ij_, where Y_ij_ is the expression level for gene i and subject j, A_j_ is the age of the subject j, and ε_ij_ is the error term. Alternative models are the above models’ subsets, including linear and quadratic ones.

The significance of the chosen regression model was estimated using the F-test, and the false discovery rate (FDR) was calculated by 1,000 random permutations of age across samples. The median of the permutation distribution was used as the null expectation. Each species was tested independently.

*Age-related genes* were defined as those showing significant age-effect in at least one species (F-test p<0.001, FDR<2% in both PFC and CBC). Genes *not* showing age-related change were defined at F-test p>0.01 in all species. Genes were defined separately for each brain region.

# Differential expression test.

The test for differential expression between a pair of species was based on analysis of covariance, or ANCOVA (Faraway 2002), and described in (Somel et al. 2009). Briefly, the test aims to identify whether two species have different curves for expression change with age, or not. First, one of the two species is selected as reference. For each age-related gene, we use the polynomial regression model chosen in the above-described age-test (for the reference species). We then test if such a regression model, but with species-specific parameters, is significantly better than the model with common parameters for both species, given the expression-age distributions of the two species. For example, in a comparison of human and chimpanzee, if the null model for gene *i* was a linear one: Y_ij_ = β_0i_ + β_1i_A_j_ + ε_ij_, we compare it to the alternative model: Y_ij_ = β_0iC_ + β_0iH_ + β_1iC_A_jC_ + β_1iH_A_jH_ + ε_ij_ , where Y_ij_ is the expression level of individual *j*, β_0iC_ and β_1iC_ are the chimpanzee-specific intercept and slope, β_0iH_ and β_1iH_ are the human-specific intercept and slope, A_jC_ and A_jC_ are chimpanzee and human ages, respectively. The null model (with no species-specific parameters) and alternative models are compared using the F-test. We test each submodel of the full alternative model, each containing one or more species-specific parameters. The differential expression test is performed on each species pair twice, using either species as a reference. For each gene, if either of the two tests was significant at a defined cutoff, we considered this gene as differentially expressed between these two species.

Permutations to estimate FDR of the difference test were performed using the 10 individual subsets per species (Figure S1a). We randomly substituted individuals of the same age-rank between two species and reapplied the test, 1,000 times for each species comparison. The results were compared with the original 10 individual subset datasets. By using the 10 individual subsets, we intended to preserve the age-structure in the data, achieve unbiased randomization of species identities. However using 10 individual subsets also cuts our sample size about half. Therefore these FDR estimates will be higher than the FDR of results using the full dataset. Also note that this FDR estimation method is conservative, as some permutations will resemble the actual constellation of species identities.

*Differentially expressed genes* were defined as those showing significant difference-test results in at least one species pair comparison (F-test p<0.001, FDR<10% in both PFC and CBC). Genes were defined in each brain region separately.

# Testing for pattern differences.

To specifically test for developmental pattern differences among species, we used the difference test on the “standardized datasets”, described above, from which constitutive expression differences had been removed. FDR was calculated using the 10 individual subsets and 1,000 permutations, as above. We defined genes showing significant developmental pattern differences as those showing significant difference-test results using the standardized datasets, in at least one species pair comparison (F-test p<0.001, FDR=18% in PFC and 34% in CBC). Genes were defined in each brain region separately. Genes *not* showing pattern difference were defined at F-test p>0.01 in all comparisons.

# Definition of three divergence types.

We defined three divergent gene sets based on the above-described tests**: Type 1 - constitutive divergence among constant genes:** genes showing constant expression (age-test p>0.01 in all 3 species) and constitutive divergence across lifespan (differential expression test p<0.001 in at least one pair of species) (n=2286 in PFC and 2704 in CBC). **Type 2 - constitutive divergence among developmental genes:** genes showing age-related change (age-test p<0.001 in min. one species) and constitutive divergence across lifespan (differential expression test p<0.001 in at least one pair of species), but no evidence for developmental pattern divergence (pattern divergence test p>0.01 in all species comparisons) (n=2761 in PFC and 2753 in CBC). **Type 3 – divergence in developmental patterns:** genes showing developmental regulation (age-test p<0.001 in at least one species) and differences in developmental expression patterns among species (both differential expression test and pattern divergence test p<0.001 in at least one pair of species) (n=1504 in PFC and 1073 in CBC).

In order to limit the false positive rate (*e.g.* an age-related gene being categorized as type 1), we used two different p–value cutoffs when defining genes showing a significant effect and genes *not* showing an effect.

Although the FDR for the difference test for pattern divergence, especially in CBC, was high, we preferred these cutoffs for two reasons: (i) Using lower p-value cutoffs could lead to high false negative rates, especially because our gene set definitions depend on multiple tests. This could limit our power to see functional differences among gene sets. (ii) Our FDR estimates are based on a limited subset of the data. Therefore our real FDR should be considerably lower than these estimates.

# Estimating expression divergence.

Expression divergence was estimated for each gene by the following procedure: (i) normalize each gene’s expression level to the same mean and variance (to allow unbiased comparisons among genes), (ii) interpolate 20 points from the expression-age trajectory, across the common age-range of the 3 species, (iii) calculate the Euclidean distance between each pair of species, (iv) use the distances to estimate a neighbor joining (NJ) tree using the R “ape” package (Paradis et al. 2004). We estimated expression divergence using (a) the full dataset and (b) the standardized dataset (constitutive differences among species removed, see above). Note that for type I and II genes (a) represents constitutive divergence; for type III genes, (b) represents developmental pattern divergence. The total NJ tree size, in turn, represents total divergence among species, while the branch lengths for each species reflect species-specific divergence. The log2-transformed ratio between human and chimpanzee branch lengths reflects the relative rate of expression differentiation in each lineage.

# Equalizing mean expression among gene sets.

Genes with higher expression levels tend to have lower noise in expression datasets, hence such genes are more likely to show statistical significance effects than others. To compare type I, II and III divergence gene sets without the influence of mean expression level, we applied the following sub-sampling procedure: (i) calculate the mean expression level in a dataset across all species, (ii) obtain the mean expression distribution of the 3 gene sets across 100 equally spaced bins (from minimum to maximum in the dataset, using the “hist” function in R), (iii) within each bin, determine the minimum number of genes among the 3 gene sets, *n*, (iv) choose *n* genes from that bin for each gene set, starting from the genes with the lowest mean expression. The procedure was applied to the PFC and CBC separately.

# Identifying species-specific genes.

For this, we used the differential expression test results, performed on each species pair twice, using either species as a reference. For each gene, if either of the two tests was significant at a defined cutoff (here differential expression test *p*<0.05) we considered this gene as differentially expressed between these two species. If a gene showed no significant differential expression between chimpanzees and macaques, but significant differential expression between humans and the other two primate species, this gene was assigned to the human-specific gene set. Chimpanzee- and macaque-specific genes were defined by analogous criteria. If a gene showed differential expression among all three species’ pairs, it was “unspecified.” For type I and II genes, we used the difference test applied on the full dataset, for type III genes, on the standardized dataset (see above).

The number of species-specific genes are shown in Figure S3e. Note the deficiency of the unspecified category among type III genes, which suggests stronger selection against expression divergence among these genes. Meanwhile, PFC type III category also shows a strong excess of human-specific genes.

# Normalizing life-history among species.

In order to normalize life-history among species, we used the following information on humans, chimpanzees and macaques: maximum age at maturity, maximum lifespan, age at first reproduction, age of eruption of first and last deciduous and permanent teeth (Smith et al. 1994; Walker et al. 2006; de Magalhães and Costa 2009). We built linear regression models using these landmarks across human-macaque and chimpanzee-macaque, treating macaque landmarks as independent variables. We transformed individual ages of human and chimpanzee using these models (*e.g.* a 98 year old human is assigned age 33 years, and a 44 year old chimpanzee is assigned age 30 years). We then repeated all analyses involving species comparisons (the differential expression test, expression divergence estimates, and gene set definitions), based on these transformed ages.

The results shown in Figure S3d and Table S3 indicate genes identified as type III drop to 60% of the originally identified numbers, and human-chimpanzee branch length ratios become ~50% shorter, but were significantly different from 1 in both PFC and CBC.

# RNA sequencing.

We prepared three human (newborn, young, old), two chimpanzee (newborn, young), and 2 rhesus macaque samples (newborn, young) using pooled total RNA from PFC and CBC of five individuals each (Table S1). RNA was isolated using Trizol® reagent (Invitrogen, Carlsbad, CA). Oligo(dT) selection was performed twice using Oligotex® mRNA Midi Kit (Qiagen). After selection, 100 ng mRNA was first fragmented by addition of 5X fragmentation buffer (200 mM Tris acetate, pH 8.2, 500 mM potassium acetate and 150 mM magnesium acetate) and heating at 94 °C for 2 min 30 s in a thermocycler, then transferred to ice and run over a Sephadex-G50 column (USA Scientific) to remove the fragmentation ions (Mortazavi et al. 2008). We used random hexamer primers (Invitrogen, Cat. No. 48190-011) for reverse transcription of fragmented mRNA to double-strand cDNA. Sequencing libraries were prepared according to the paired-end sample preparation protocol ([http://www.illumina.com](http://www.illumina.com/)). Each sample was sequenced in a separate lane in the Illumina® Genome Analyzer II system, using the 75-bp paired-end sequencing protocol.

# RNA-sequencing analysis.

The raw sequence reads were mapped to the reference genomes (hg19, panTro2, and rheMac2), allowing a maximum of four mismatches, using the “Tophat” program (<http://tophat.cbcb.umd.edu/>) (Trapnell et al. 2009). Only uniquely mapped reads were used in downstream analysis. Human gene annotation was downloaded from Ensembl (v56; http://www.ensembl.org). Chimpanzee and rhesus macaque gene annotations were constructed from human gene annotation using the UCSC “Liftover” tool (<http://www.genome.ucsc.edu/cgi-bin/hgLiftOver>). Gene annotations were filtered to only include transcripts with similar size in all three species (*i.e.* the difference in transcript length between species is less than the length of the shorter transcript). Normalized transcript expression levels were calculated using the “Cufflinks” program (http://cufflinks.cbcb.umd.edu/), employing the mapping result from “Tophat” and conserved exon annotation from “Liftover” software (Trapnell et al. 2010). If a gene contained more than one transcript, the expression value of the longest one was chosen as representative. The processed datasets are available at the following locations for PFC and CBC, respectively:

<http://www.picb.ac.cn/Comparative/data_methods/age_divergence_2010/rnaseq_hcm_PFC1.txt> <http://www.picb.ac.cn/Comparative/data_methods/age_divergence_2010/rnaseq_hcm_CBC1.txt>

For comparing divergence types using the RNA-seq datasets, we used the following approach: (i) for each gene, calculate expression differences between human newborn and macaque newborn, and human young adult and macaque young adult, (ii) sum the absolute value of these differences, (iii) repeat the steps i-ii with chimpanzee and macaque, (iv) compare the human-macaque distance (*h-m*) and chimpanzee-macaque distance (*c-m*) among a set of genes, assuming that *h-m*>*c-m* indicates excess human-specific changes. This procedure was applied to (a) the full RNA-seq dataset (without human old adult samples), (b) standardized RNA-seq dataset (without human old adult samples, after standardizing expression levels among species to mean=0 and s.d.=1). Upon standardization, ~1/3 of genes showed *h-m*=*c-m*, and were ignored in subsequent analysis. Finally, we compared *h-m* and *c-m* distances obtained from the RNA-seq dataset across the 3 divergent gene types identified in the microarray analysis. The results are shown in Figure S4.

# Sequence divergence estimates.

For estimating divergence in protein coding sequence, we used human-mouse and human-macaque dN/dS ratios downloaded from Ensembl (v55). For estimating promoter conservation, we used the Phastcons 18-way Placental Mammal Conservation Track (a subset of the 28-way Placental Track) from the UCSC Genome Browser (Siepel et al. 2005). For each Ensembl human gene, we computed mean PhastCons score per proximal promoter (+/- 200 bp around the transcription start site). Using 2,000 bp yielded similar results (data not shown). Similarly, we calculated mean Phastcons scores across the 3’UTR of each Ensembl gene. For genes with multiple isoforms, we chose the one with largest number of exons.

Pan-mammal sequence divergence measures are negatively correlated with mean brain gene expression levels (r~0.2-0.3). To control for this, when comparing different gene sets with respect to sequence divergence, we only used gene subsets with equalized mean expression levels distributions (see above). Using an alternative approach, removing the expression level effect by using residuals from linear regression models between divergence and mean expression, yielded the same results (data not shown).

# Identification of species-specific mutations in regulatory regions.

Human gene annotation was downloaded from Ensembl (v54). For each Ensembl human gene, we extracted the proximal promoter (+/-1 kb upstream and downstream of the TSS) and 3’UTR regions from the human genome (hg18). We further projected these coordinates onto chimpanzee (panTro2) and rhesus macaque (rheMac2) genomes using the “Liftover” tool and Pairwise Alignments downloaded from UCSC Genome Browser (Karolchik et al. 2008). The minimum ratio of remapped bases were set to 0.8 via the minMatch parameter in the “Liftover” program. We thus obtained orthologous promoter sequences across the three species. Multiple alignments were done using the MUSCLE program (MUSCLE v3.7, <http://www.drive5.com/muscle>). Based on these alignments, mutations observed in one of the 3 species (*i.e.* specie-specific mutations) were identified using custom Python scripts. As inflated mutation estimates can arise due to homology or genome alignment problems, we removed the 5% upper fraction of mutation estimates.

Notably, we did not observe any gross excess of regulatory sequence divergence in any of the three lineages, as observed among type III genes’ expression divergence (data not shown).

We did not correct for multiple testing for these sequence-expression divergence correlations, as the sequence divergence estimates are not fully independent.

# Functional enrichment analysis.

Kyoto Encylopedia of Genes and Genomes (KEGG) pathway annotation (Kanehisa et al. 2008) and Gene Ontology (GO) annotation (Ashburner et al. 2000) for human were downloaded from KEGG (http://www.genome.jp/kegg/) and Ensembl (v54) databases, respectively. We used the GO “term” and “graph path” tables downloaded from the GO database (http://archive.geneontology.org/latest-termdb/) to associate each GO term with higher terms. To identify overrepresented KEGG pathways or GO terms, we used the hypergeometric test, and adjusted *p*-values for multiple-testing by Bonferroni correction. To restrict the search space *a priori*, only pathways/terms containing ≥5 genes were tested.

# Expression breadth and cell type-specificity analyses.

To estimate expression breadth per gene, we used information on how many cell types (epithelium, endothelium, hepatocyte, melanocyte, B-lymphoblast, T-lymphoblast) a gene was expressed in, based on the GNF dataset available from Ensembl (v54) (Hubbard et al. 2007). Alternatively, using the number of organ systems a gene was expressed in, based on the same GNF dataset, produced comparable results (data not shown). For estimating the neural-specificity of a gene, we used two data sources. (a) We obtained all genes containing the terms “neuron”, “axon” and “synapse” from GO and linked these to Ensembl human genes, which resulted in 1715 genes. These were compared to 14515 genes with GO information but not linked to neuronal functions. (b) We used expression levels measured in purified mouse neurons, astrocytes and oligodendrocytes (Cahoy et al. 2008). Data was preprocessed as described in (Somel et al. 2010). We obtained human-mouse one-to-one orthologs using Ensembl (v54) and defined neuron-, astrocyte-, oligodendrocyte- and oligodendrocyte-precursor-related genes, using an effect size cutoff Cohen’s d>2. From these sets we further defined 1116 neuron-related, 2490 glia-related and 5687 non-specific genes. We controlled for expression level-expression breadth/specificity correlations by using gene subsets with equalized mean expression levels distributions (see above). Removing the expression level effect by using residuals from linear regression models between breadth/specificity and mean expression yielded the same results (data not shown).

# MicroRNA and TF target identification.

The procedure to define conserved human miRNA and TFBS target sites is described in (Somel et al. 2010). For miRNA target prediction we used the Conserved Site Context Score Table from TargetScan (v5.0) (Lewis et al. 2005). Conserved TF binding sites (TFBS) were estimated as follows: Promoter regions (+/-2,000 bp around the transcription start site) for each gene were extracted based on the gene annotation from Ensembl (v54). Predicted TFBS in each promoter were identified using the Match™ algorithm, employing the TFBS sequences from the TRANSFAC® database (Kel et al. 2003) (v11.2). The average Phastcons scores of each predicted TFBS were calculated using the UCSC Genome Browser 17-way vertebrate Conserved Element Table (Siepel et al. 2005). We defined conserved TFBS as those for which ≥80% of nucleotides had Phastcons scores and the average score was ≥0.6. TFBS were linked to transcription factors’ Ensembl gene IDs using two paths: (a) HGNC symbols were parsed from the Transfac® Matrix Table (v11.2) and mapped to Ensembl genes via HGNC ([www.genenames.org/data/gdlw_index.html](http://www.genenames.org/data/gdlw_index.html)). (b) TRANSFAC TFBS IDs and corresponding SwissProt IDs were extracted from the UCSC Genome Browser HMR Conserved Transcription Factors Table and mapped to Ensembl genes. Gene lists from the two paths were combined, resulting in 426 TFBS mapped to ≥1 gene that were annotated with “transcription factor activity” (GO:0003700) in Ensembl. For analysis presented in Fig. 3a, we removed the overall sequence conservation effect on BS density, by using residuals from linear regressions between TFBS/miRNA-BS density and promoter/3’UTR mean Phastcons scores, respectively.

# MicroRNA microarrays.

Total RNA was extracted from dissected CBC and PFC samples (humans, n=14; chimpanzees, n=11; macaques, n=8, respectively; Table S1), using the Ambion® mirVana miRNA isolation kit. For each sample, 100 ng isolated RNA were hybridized to Agilent® Human microRNA Microarray (v3), which contains probes for 866 human and 89 human viral microRNAs based on the Sanger miRBase Release 12.0. MicroRNA labeling, hybridization and washing were performed according to manufacturer's instructions (López-Romero et al. 2010). For human and chimpanzee, the microarray experiments were carried out in two batches. Two individuals in human PFC and CBC sets and 5 individuals in chimpanzee PFC and CBC sets were measured in both batches to estimate the technical variance. Arrays were scanned by a DNA microarray scanner (Agilent G2565BA). The Agilent G4462AA Feature Extraction software (v10.5.1.1) was used for image analysis with default settings. We included only samples with detected miRNAs more than 200, thus removing samples with overall weak hybridization signals. In total, 8 human, 11 chimpanzee, and 8 macaque PFC samples and 12 human, 7 chimpanzee, and 7 macaque CBC samples were retained for downstream analysis. In each brain region, miRNAs that could be detected in at least half the samples in each species were used. The data was log_2_ transformed and quantile normalized. We further removed any batch effects by equalizing the mean expression level across batches. As the miRNA microarray probes are designed based on human mature miRNA sequences, we further removed miRNAs that showed any sequence difference in their mature sequences. For this, we first extracted miRNA precursor sequences in chimpanzee and macaque genomes (panTro3 and rheMac2, respectively) corresponding to human precursors using BLAT (Kent 2002) and then aligned these by ClustalW2 (http://www.ebi.ac.uk/Tools/msa/clustalw2) to extract the orthologous mature sequences. 500 human miRNAs showing 100% conservation across the three species were included in our downstream analysis. All primary data is deposited in the NCBI Gene Expression Omnibus database (GEO; <http://www.ncbi.nlm.nih.gov/geo/>) (ID number pending). The processed datasets are available at the following locations for PFC and CBC, respectively:

<http://www.picb.ac.cn/Comparative/data_methods/data_ms_mirna_age_pfc_2011.txt>

<http://www.picb.ac.cn/Comparative/data_methods/data_ms_mirna_age_cbc_2011.txt>

We applied to the miRNA datasets the same age and differential expression tests applied to mRNA. However, due to the smaller sample size of the miRNA datasets, using the same significance cutoffs (F-test p<0.001) to define divergent gene types yielded too few miRNAs per type (data not shown). We therefore used F-test p<0.01 as a cutoff for the age, differential expression and pattern difference tests.

# Regulator-target correlations.

We tested the overall effect of regulator (miRNA or TF) expression variation on predicted targets’ expression variation in a specific gene set, such as type III genes, as described in (Somel et al. 2010), which is as follows: (i) Calculate the correlations between each regulator and its predicted targets, using data from all 3 species, (ii) calculate the relative frequency of Pearson correlation coefficients within a gene set, using the Gaussian kernel density from the R “density” function, (iii) calculate the background distribution, *i.e.* the kernel densities for the correlations between regulators and genes which were not predicted to be their targets (non-targets). Note that non-targets will be predicted targets of other regulators. (iv) Calculate the difference between the kernel density distributions of regulator-target correlations and regulator-non-target correlations (represented in Fig. 3e-f). (v) Estimate the excess of negative or positive correlations for miRNA and TF targets, respectively, by comparing the total number of correlated targets, with correlated non-targets. For this we used cutoffs r<-0.9 and r>0.9, as well as r<-0.75 and r>0.75 and used the hypergeometric test (Table S6). (vi) For Fig. 3, we randomly sampled from the regulator-non-target pairs (*i.e.* background), the same number as regulator-target pairs, applied steps (i-iv), and repeated this 100 times.

Regulator-target correlations may be influenced by both age and species effects. As our main focus is on regulators of expression divergence among species, we used a second approach: (i) For each gene and regulator, and for each species, interpolate 20 points across the three species’ common age-range (see above). (ii) Calculate the differences between a pair of species using these interpolated points. (iii) Concatenate the three sets of species differences (one per species pair) for each gene. (iv) Calculate regulator-target correlations based on these species differences.

These comparisons indicated miRNA and TF effects on the expression of different gene types in both brain regions (Table S6). Notably, the only instance where miRNA affected age-related changes and species differences simultaneously was PFC type III genes.

# Identification of candidate regulators.

For identifying candidate regulators of a specific gene set, such as type III genes, we used the procedure described in (Somel et al. 2010), as follows: (i) For each candidate regulator (here miRNA), test enrichment of predicted targets in that gene set, compared to all other gene sets (*e.g.* type I and II genes), using a one-sided hypergeometric test (HT). Here, using all expressed genes as background yielded similar results (data not shown). (ii) Estimate the random expectation for this test by permuting genes among gene sets 1000 times and repeating the test. (iii) If a regulator’s targets are enriched among PFC type III genes at HT p<0.05, consider that regulator “specific” to PFC type III genes. Consider all other regulators (at HT p>0.05) “non-specific.” (iv) For each “specific” regulator, calculate the correlation between its expression profile and its PFC type III targets’ expression profiles. (v) Calculate the proportion of highly negatively correlated (for miRNA, at r<-0.75) targets among this target set, *P*. (vi) Calculate the same proportion for each “non-specific” regulator and its targets among PFC type III genes. We assume these to represent the background distribution, and the mean of these proportions to represent the random expectation. (vii) Compare *P* of each “specific” regulator with the mean *P* among non-specific regulators, using a one-sided binomial test.

A putative regulator was defined as one with an enrichment of targets in a gene set (HT p<0.05) and with an excess of correlations with those targets (BT p<0.05). For each candidate regulator, steps iv-vii were repeated, first using correlations based on expression profiles and second using correlations based on species differences (see above section).

# Verified miRNA target lists and functional enrichment tests.

Verified targets lists for each of the top three miRNAs were defined as genes (a) being identified as targets of the miRNA in TargetScan, (b) showing negative correlation with the miRNA (r < -0.75) based on the time series (either using the full trajectories, or species differences, respectively), (c) showing negative expression change upon transfection in the cell line experiment. Note that the transfection experiment independently supported miRNA-target associations both predicted by TargetScan, and predicted by the time-series (except for miR-92a showing a non-significant excess of negative correlations in Fig 4C).

For functional analysis of miRNA targets, we used the same approach as described above, but chose a cutoff of ≥2 genes per KEGG set. In addition, we used all expressed miRNA targets as background.

For estimating the probability of finding the same KEGG group enriched between two miRNA target sets, we used the following randomization procedure: We randomly sampled two gene sets from all PFC type III genes annotated by TargetScan. The sets had the same number of genes as miR-454 and miR-92a verified targets (n=18 and n=20, respectively), with at least one gene overlapping between the sets, as one gene indeed overlapped between the two sets. For 20,000 times, we conducted the KEGG pathway enrichment test (see above) for both sets. The probability that both sets had at least marginal significance (Bonferroni corrected p-value<0.10) was p=0.032. Conditioning on one set being marginally significant, the probability that the same KEGG pathway was enriched in both sets was p=0.021.

# Analysis of mouse developmental series.

We analyzed a mouse postnatal brain development dataset comprising 18 individuals from two mouse species, *Mus musculus* and *Mus spretus* (Somel et al. 2009). Individuals had been sampled at 0, 14 and 56 days. The genomic divergence levels of these species are on a par with human-chimpanzee divergence (Enard et al. 2002). Probes showing affinity differences between species were excluded as described in (Somel et al. 2009). The same age- and differential expression tests and p-value cutoffs were used as described above, and mouse Ensembl genes were sorted into the three types of expression divergence, as in the primate analyses. The total number of differentially expressed genes identified in the mouse dataset (n=1664) was similar to that identified between human and chimpanzee in PFC (n=2367) or CBC (n=1996). Specifically, such genes comprised <1% of all significantly differently expressed genes between the two species. In comparison, 7% and 15% of divergent genes showed developmental divergence in human-chimpanzee comparisons in the CBC and PFC, respectively.

# Cell line transfections.

We transfected two human neuroblastoma cell lines, SH-SY5Y and SK-N-SH, with three miRNA constructs containing mature sequences for miR-92a (5’-UAUUGCACUUGUCCCGGCCUGU-3’), miR-454 (5’-UAGUGCAAUAUUGCUUAUAGGGU-3’) and miR-320b (5’-AAAAGCUGGGUUGAGAGGGCAA-3’), as well as two negative controls with sequences 5’-UUCUCCGAACGUGUCACGUTT-3’ and 5’-ACGUGACACGUUCGGAGAATT-3’. Constructs were designed based on Mirbase (Griffiths-Jones et al. 2006).

One day before transfection, cells were plated in growth medium (DMEM (Hyclone) + 10% FBS (Hyclone) medium) without antibiotics in a 24-well plate until 30-50% confluence. We prepared miRNA construct-Lipofectamine™ 2000 (Invitrogen, Cat. No. 11668-027) complexes freshly before transfection according to the manufacturer’s instructions. Each well of cells was added 100μl complex and kept at 37°C in a CO2 incubator for 4-6 hours. The medium was replaced with complete medium and culture for next 20-24 hours.

Total RNA was labeled and hybridized to ten Affymetrix HG U133Plus2.0 arrays following standard protocols. For each of the two cell lines, we processed five samples: One for each miRNA and 2 negative controls. We processed the Affymetrix .CEL files using the R “affy” library, summarizing expression levels using the CustomCDF (v12) files for Ensembl genes downloaded from (http://brainarray.mbni.med.umich.edu/Brainarray/Database/CustomCDF/CDF_download.asp) (Dai et al. 2005). We first removed any mean cell line effects by subtracting the mean of each cell line per gene from all 5 arrays per cell line. For each miRNA, for each cell line, and for each gene, we subtracted the mean expression in the negative control samples from the miRNA transfected sample. The quantile normalized and log2 transformed data can be found at:

<http://www.picb.ac.cn/Comparative/data_methods/age_divergence_2010/hsNeubMir92a_Mir320b_Mir454_NC.tsv>

# *In situ* hybridizations.

Hybridizations were performed following (Silahtaroglu et al. 2007). Tissue sections were collected on Superfrost/plus slides (Fisher). For fixation of proteins and nucleic acids in tissues, a solution containing 4% formaldehyde was used. Formaldehyde protein cross-links, and nucleic acid base modifications, were reverted by treatment with proteinase K, which can weaken formalin cross-links (Masuda et al. 1999). After washing in two changes of excess PBS, sections were acetylated in 0.1M triethanolamine/0.25% acetic anhydride for 10 minutes and then incubated in humidified bioassay trays for prehybridization at 50°C (20-25°C below the Tm of the probe) for 4 hours in hybridization buffer (5xSSC/lx Denhardt's solution/5 mM EDTA/0.1% Tween/0.1% DHAPS/50% deionized formamide/0.1 mg/ml Heparin and 0.3 mg/ml yeast tRNA) (Jankowsky et al. 2000; Silahtaroglu et al. 2007). This was followed by an over-night hybridization step using a DIG-labeled LNA oligonucleotide probe complementary to the target miRNA. Under the temperature of 50°C, sections were rinsed and washed twice in 2xSSC and 3 times in 0.2xSSC. Nonspecific binding could be reduced by more stringent washing conditions or by an intense RNase A digestion (10 mg/ml). The in situ signal was detected by incubation with alkaline phosphatase (AP)-conjugated anti-DIG antibody, using NBT/BCIP as substrate.

Supporting References

Ashburner M, Ball C, Blake J, Botstein D, Butler H et al. (2000) Gene Ontology: tool for the unification of biology. Nat Genet 25(1): 25-29.

Boedigheimer MJ, Wolfinger RD, Bass MB, Bushel PR, Chou JW et al. (2008) Sources of variation in baseline gene expression levels from toxicogenomics study control animals across multiple laboratories. BMC Genomics 9: 285.

Cahoy JD, Emery B, Kaushal A, Foo LC, Zamanian JL et al. (2008) A transcriptome database for astrocytes, neurons, and oligodendrocytes: a new resource for understanding brain development and function. J Neurosci 28(1): 264-278.

Cheung I, Shulha HP, Jiang Y, Matevossian A, Wang J et al. (2010) Developmental regulation and individual differences of neuronal H3K4me3 epigenomes in the prefrontal cortex. Proc Natl Acad Sci U S A 107(19): 8824-8829.

Clancy B, Darlington R, Finlay B (2001) Translating developmental time across mammalian species. Neuroscience 105(1): 7-17.

Dai M, Wang P, Boyd A, Kostov G, Athey B et al. (2005) Evolving gene/transcript definitions significantly alter the interpretation of GeneChip data. Nucl Acids Res 33(20): e175-.

de Magalhães JP, Costa J (2009) A database of vertebrate longevity records and their relation to other life-history traits. J Evol Biol 22(8): 1770-1774.

Enard W, Khaitovich P, Klose J, Zollner S, Heissig F et al. (2002) Intra- and Interspecific Variation in Primate Gene Expression Patterns. Science 296(5566): 340-343.

ENCODE_Project_Consortium, Myers RM, Stamatoyannopoulos J, Snyder M, Dunham I et al. (2011) A user's guide to the encyclopedia of DNA elements (ENCODE). PLoS Biol 9(4): e1001046.

Faraway J (2002) Practical Regression and Anova Using R.

Gautier L, Cope L, Bolstad B, Irizarry R (2004) affy--analysis of Affymetrix GeneChip data at the probe level. Bioinformatics 20(3): 307-315.

Griffiths-Jones S, Grocock R, van Dongen S, Bateman A, Enright A (2006) miRBase: microRNA sequences, targets and gene nomenclature. Nucl Acids Res 34(suppl_1): D140-144.

Hubbard T, Aken B, Beal K, Ballester B, Caccamo M et al. (2007) Ensembl 2007. Nucl Acids Res 35(suppl_1): D610-617.

Jankowsky JL, Derrick BE, Patterson PH (2000) Cytokine responses to LTP induction in the rat hippocampus: a comparison of in vitro and in vivo techniques. Learn Mem 7(6): 400-412.

Kanehisa M, Araki M, Goto S, Hattori M, Hirakawa M et al. (2008) KEGG for linking genomes to life and the environment. Nucleic Acids Res 36(Database issue): D480-484.

Karolchik D, Kuhn RM, Baertsch R, Barber GP, Clawson H et al. (2008) The UCSC Genome Browser Database: 2008 update. Nucleic Acids Res 36(Database issue): D773-779.

Kel AE, Gössling E, Reuter I, Cheremushkin E, Kel-Margoulis OV et al. (2003) MATCH: A tool for searching transcription factor binding sites in DNA sequences. Nucleic Acids Res 31(13): 3576-3579.

Kent W (2002) BLAT---The BLAST-Like Alignment Tool. Genome Res 12(4): 656-664.

Khaitovich P, Hellmann I, Enard W, Nowick K, Leinweber M et al. (2005) Parallel Patterns of Evolution in the Genomes and Transcriptomes of Humans and Chimpanzees. Science 309(5742): 1850-1854.

Kim T-K, Hemberg M, Gray JM, Costa AM, Bear DM et al. (2010) Widespread transcription at neuronal activity-regulated enhancers. Nature 465(7295): 182-187.

Lewis BP, Burge CB, Bartel DP (2005) Conserved seed pairing, often flanked by adenosines, indicates that thousands of human genes are microRNA targets. Cell 120(1): 15-20.

Li J, Bushel P, Chu T-M, Wolfinger R (2009) Principal Variance Components Analysis: Estimating Batch Effects in Microarray Gene Expression Data. In: A S, editor. Batch Effects and Noise in Microarray Experiments: Sources and Solutions. Chichester: John Wiley & Sons, Ltd. pp. 141-154.

López-Romero P, González MA, Callejas S, Dopazo A, Irizarry RA (2010) Processing of Agilent microRNA array data. BMC Res Notes 3: 18.

Lu L, Airey D, Williams R (2001) Complex trait analysis of the hippocampus: mapping and biometric analysis of two novel gene loci with specific effects on hippocampal structure in mice. J Neurosci 21(10): 3503-3514.

Masuda N, Ohnishi T, Kawamoto S, Monden M, Okubo K (1999) Analysis of chemical modification of RNA from formalin-fixed samples and optimization of molecular biology applications for such samples. Nucleic Acids Res 27(22): 4436-4443.

Maycox PR, Kelly F, Taylor A, Bates S, Reid J et al. (2009) Analysis of gene expression in two large schizophrenia cohorts identifies multiple changes associated with nerve terminal function. Mol Psychiatry 14(12): 1083-1094.

Mortazavi A, Williams BA, McCue K, Schaeffer L, Wold B (2008) Mapping and quantifying mammalian transcriptomes by RNA-Seq. Nat Methods 5(7): 621-628.

Paradis E, Claude J, Strimmer K (2004) APE: Analyses of Phylogenetics and Evolution in R language. Bioinformatics 20(2): 289-290.

Shupe J, Kristan D, Austad S, Stenkamp D (2006) The eye of the laboratory mouse remains anatomically adapted for natural conditions. Brain Behav Evol 67(1): 39-52.

Siepel A, Bejerano G, Pedersen JS, Hinrichs AS, Hou M et al. (2005) Evolutionarily conserved elements in vertebrate, insect, worm, and yeast genomes. Genome Res 15(8): 1034-1050.

Silahtaroglu AN, Nolting D, Dyrskjøt L, Berezikov E, Møller M et al. (2007) Detection of microRNAs in frozen tissue sections by fluorescence in situ hybridization using locked nucleic acid probes and tyramide signal amplification. Nat Protoc 2(10): 2520-2528.

Smith BH, Crummett TL, Brandt KL (1994) Ages of eruption of primate teeth: A compendium for aging individuals and comparing life histories. Am J Phys Anthropol 37(S19): 177-231.

Sokal RR, Rohlf FJ (1995) Biometry. New York: Freeman.

Somel M, Franz H, Yan Z, Lorenc A, Guo S et al. (2009) Transcriptional neoteny in the human brain. Proc Natl Acad Sci USA 106(14): 5743-5748.

Somel M, Guo S, Fu N, Yan Z, Hu HY et al. (2010) MicroRNA, mRNA, and protein expression link development and aging in human and macaque brain. Genome Res 20: 1207-1218.

Trapnell C, Pachter L, Salzberg SL (2009) TopHat: discovering splice junctions with RNA-Seq. Bioinformatics 25(9): 1105-1111.

Trapnell C, Williams BA, Pertea G, Mortazavi A, Kwan G et al. (2010) Transcript assembly and quantification by RNA-Seq reveals unannotated transcripts and isoform switching during cell differentiation. Nat Biotechnol 28(5): 511-515.

Walker R, Burger O, Wagner J, Von Rueden C (2006) Evolution of brain size and juvenile periods in primates. J Hum Evol 51(5): 480-489.
